# Fertigation with Zn-Lysine Confers Better Photosynthetic Efficiency and Yield in Water Stressed Maize: Water Relations, Antioxidative Defense Mechanism and Nutrient Acquisition

**DOI:** 10.3390/plants11030404

**Published:** 2022-02-01

**Authors:** Faisal Shehzad, Qasim Ali, Shafaqat Ali, Fahad A. Al-Misned, Saliha Maqbool

**Affiliations:** 1 Department of Botany, Government College University, Faisalabad 38000, Pakistan; shahzadrana543@gmail.com; 2 Department of Environmental Sciences and Engineering, Government College University, Allama Iqbal Road, Faisalabad 38000, Pakistan; 3 Department of Biological Sciences and Technology, China Medical University, Taichung 40402, Taiwan; 4 Department of Zoology, College of Science, King Saud University, Riyadh 11451, Saudi Arabia; almisned@ksu.edu.sa; 5 Department of Soil, Water, and Climate, University of Minnesota, Saint Paul, MN 55108, USA; smaqbool@umn.edu

**Keywords:** amino acids, antioxidants, chelation, grain yield, lysine, micro-nutrients, photosynthesis, zinc

## Abstract

Different strategies including the exogenous use of micronutrient-chelated amino acids are being employed for better crop yield with limited fresh water for irrigation. The present study was conducted to assess the effects of foliar-applied Zn-lysine (Zn-Lys) on maize growth and yield under limited irrigation, in relation to physio-biochemical mechanisms such as the plant–water relations, photosynthetic efficiency, antioxidant defense mechanism, amino acid accumulation and nutrient acquisition. The experiment comprised two maize cultivars (MMRI and Pearl), two irrigation levels and three levels of Zn-Lys (0.25, 0.5 and 0.75%). Zn-Lys fertigation was found to be effective in reducing the negative impacts of limited water supply on grain yield, associated with improved photosynthetic efficiency, water relations, antioxidative defense mechanism and reduced lipid peroxidation in both maize cultivars. Zn-Lys-induced improvement in antioxidative mechanisms was associated with improved content of non-enzymatic antioxidants and activities of antioxidant enzymes. Foliar-fertigation with Zn-Lys also significantly improved the contents of various amino acids including Lys, as well as uptake of nutrients in both maize cultivars. In conclusion, the 0.5% level of Zn-Lys was found to be effective in ameliorating the negative impacts of water stress for better grain yield in both maize cultivars that can also be used as an important environment-friendly source of Zn to fulfill maize Zn deficiency.

## 1. Introduction

In crop plants, limited fresh water for irrigation along with abrupt changes in the environment are major problems at present for obtaining better seed yields [1]. This reduction in seed yield due to water shortage is also linked with disturbances in soil physio-chemical properties that alter the biochemical and physiological activities of plants [2,3,4]. Water shortage reduces the bioavailability of nutrients in the rhizospher for uptake through roots due to a decrease in soil osmotic potential [5,6,7]. This results in disturbed plant–water relations, plant photosynthetic efficiency and antioxidative defense mechanisms, leading to decreased growth and seed yield [6,7,8,9]. Under such conditions, the soil nutrient status is of prime importance [10]. Therefore, it is necessary to maintain a balance in plant nutrient status for better plant functioning, especially under water deficit conditions [11]. Nutrients, either macro- or micro-, play significant roles in obtaining better crop yields, especially under stressful environments [12]. It is also well understood that nutrients play significant roles in plant stress tolerance, including under water shortage conditions [13,14,15]. However, the problem is intensified when the nutrient deficiency already prevails in cultivated soil. In view of the roles of different essential micro-nutrients, zinc (Zn) has key roles in crop plants in obtaining better seed yield as well as in plant stress tolerance under different abiotic stresses [16,17,18]. Its deficiency also causes malnutrition in humans and in animals [19]. It is well understood that most of the world’s cultivated lands are deficit in Zn bioavailability. The concentration of Zn is low in semi-arid and arid regions of world due to its low solubility and high fixation under such conditions [20,21]. In Pakistan, about 70% of agricultural land is Zn deficit [22], which has caused the problem of Zn malnutrition in consumers living in these areas and being dependent on the productivity of the crops grown in these areas [23]. It has been found that the Zn deficient soils not only decrease the crop production but also negatively affect the seeds’ nutritional quality which has created the problem of Zn malnutrition, leading to the poor mental growth, especially in infants [19,24]. Among common crops, the Zn deficit soils have large impacts on various biological aspects of plants, especially on the yield of cereals, including maize, and the world depends on the productivity of cereal crops to fulfill food demands [25]. At present the Zn availability in agricultural soils is considered an important issue under water deficiency due to its reduced uptake [26]. 

At the global level, 47% of agricultural land is water deficit and this is being further threatened by changing environmental conditions, especially due to global warming and altered rainfall patterns [27]. In Pakistan about 25% of the area is rainfed [28] and is expected in the near future to face severe water deficiency [29]. Therefore, under such conditions, the use of new strategies is necessary to counteract such problems for better seed yield under water deficit conditions. Different strategies including exogenous use of different chemicals through different modes are being employed for better crop production. It has been shown that, plants grown in Zn deficit soils show stunted stem growth and symptoms of leaf chlorosis [30]. Zinc deficiency adversely affects the plant photosynthetic efficiency due to decreased intercellular CO_2_ concentration by negatively affecting the stomatal conductance [31,32,33]. The negative impacts of Zn deficiency on leaf photosynthetic efficiency are also associated with reduced activity of carbonic anhydrase enzyme [34]. Zinc deficiency also disturbs plant cellular water relations by effecting the osmotic adjustment [33,35] that directly effects the transpiration rate and photosynthesis. In chickpea, grain yield was improved significantly due to exogenous application of Zn under well-watered conditions [36]. In wheat, exogenous application of Zn improved the plant photosynthetic rate when grown under limited water supply [37].

Zinc has significant effects on cell expansion, cell division, carbohydrate, protein, nucleic acid biosynthesis as well as regulating lipid metabolism [38]. At the cellular level Zn also controls auxin metabolism, a well-known essential plant growth regulator [39]. Deficiency of Zn results in slowing down the process of transcription as well as deformation and reduction in ribosomes [40,41]. Regarding its stress reducing impacts, especially under water stress, Zn application improves the uptake of metal ions through the activation of metal transcription factor (TFs) by directly binding to it [42]. Exogenous application of Zn also affects the water uptake by roots and then its upward movement [43]. Independently Zn also plays important role in maintaining the antioxidative defense mechanism by acting as a cofactor of many antioxidative enzymes, including glutathione peroxidase and superoxide dismutase [44,45]. It has been found that Zn-finger transcription factor maintains the structure of many different proteins [46,47]. Due to the significant role of Zn for better plant growth along with better production, varying techniques are being used to overcome plants Zn deficiency that include the exogenous use of different Zn fertilizers through different modes [48,49,50]. These include the use of ZnSO_4_, ZnO, Zn-EDTA, Zn-oxosulfate and Zn-liginosulfate [51,52]. The most used Zn fertilizer is ZnSO_4_ [53,54,55]. All these Zn sources are inorganic in nature and have been found to be toxic to some extent for the environment, plants and consumers [56,57]. Recently, interest has been increasing in the use of fertilizers that are organic in nature, including the amino acid-chelated metal ions that are considered environmentally friendly and non-toxic [35] as well as being helpful in improving seed germination [35,58], plant growth and seed nutritional quality by better nutrient uptake and its translocation under stressful conditions [59,60].

In view of the literature reviewed, it was hypothesized that exogenously applied Zn-Lys might be helpful for drought tolerance induction by improving the plant Zn and Lys of maize. Second, it might also be helpful in improving the grain nutritional quality of maize plants under water deficit stress, as it is well known that maize crops have the problem of Zn deficiency all over the world, compared with other crops [61]. Moreover, the maize grain is deficient in lysine due to lysine deficiency during grain filling. Lysine, as an essential amino acid, has significant roles in signaling cascades during environmental stresses. It has also been shown that lysine is involved in gamete production, fertilization, and seed development [62]. Maize is a major cereal crop all over the world, along with wheat and rice, to fulfill world food demand [63]. In Pakistan, it is grown over an area of 1016 Mha [63] and most of the areas under maize production in Pakistan are rainfed [64]. The aim of the present study is to induce drought tolerance in maize by foliar application of Zn-Lys, focusing the biomass production and yield of plants, by considering plant photosynthetic efficiency, water relations, biochemical and physiological parameters, nutrient uptake patterns and the antioxidative defense mechanism.

## 2. Materials and Methods

The present experiment was performed in the field under natural conditions in the New Botanical Garden of Government College University Faisalabad. Two high yielding maize genotypes (Pearl and MMRI) were used for the experimentation with germination potential of more than 90%. The seeds of both maize genotypes were purchased from Maize and Millet Research Institute, Yousafwala, Sahiwal, Pakistan. For confirmation of results, the experiment was conducted in two consecutive years during 2019 and 2020 in maize growth season. 

### 2.1. Experimental Layout

The whole experimental area was arranged in a split plot arrangement. The area allocated for the experiment was divided into two main plots corresponding to each irrigation regime. Each main plot was then divided into five subplots, one for each specific treatment. The subplots comprised four rows of equal size considered as the replicates, with a 75 cm row to row distance. First irrigation to each main plot was applied 15 days before seed sowing. The soil was well prepared by ploughing when the soil was at field capacity and supplied with adequate amount of fertilizer [N (160 Kg/ha), P (80 Kg/ha) and K (50 Kg/ha)] as per recommendations. 

### 2.2. Sowing of Seeds and Water Stress Treatment

The rate of seed sowing was 10 kg/ha^−1^ for each maize cultivar. The seeds were hand sown in furrows. Seed sowing was done in the month of July in two consecutive years during 2019 and 2020. Eight days after the emergence of seedling, to maintain the plant-to-plant distance of 30 cm, thinning was carried out. After the thinning, all plots were irrigated. After 15 days of seedling emergence, water deficit stress treatment was started by controlling the irrigation schedule. To the non-stressed plots, irrigation was scheduled every 15 days as per the water requirements, while the irrigation to the plots allocated as water deficit treatment was scheduled every 21 days. 

### 2.3. Foliar Application of Zn-Lys

Fifteen days after of the start of water stress treatment, different levels of Zn-Lys were supplied as a foliar treatment at the early vegetative stage. Different treatments were 0.25, 0.5 and 0.75% levels of Zn-chelated lysine along with water spray (WS) and no spray (NS) treatment. Tween-20 (0.1%) was added to each prepared solution as a surfactant. The evening time before sunset was chosen for spray for the maximum absorption of applied solution. An aliquot of 500 mL was supplied to plants in each replicate using manually operated agricultural spray equipment with an 8 L tank capacity fitted with a brass spray lance with a fine spray brass nozzle and a pump barrel made of seamless brass tube.

### 2.4. Preparation of Zn-Lys Chelation

Zn-lysine was prepared using the method of Leu [65]. The Zn-lysine chelation was prepared by dissolving ZnSO_4_·7H_2_O and lysine monohydrochloride (1:2) in 150 mL of distilled water with continuous heating and stirring at 95 °C for 1 h. The mixture was then dried, stored at 4 °C and used later for further experimentation.

### 2.5. Soil Analysis

The soil texture of the experimental area was sandy clay with average of 65% clay, 22% sand and 13% silt. The other properties of the soil were as follows: organic matter content, 0.78%, saturation percentage of soil, 31 [66]; NO_3_–N 6.5, NH_4_–N 3.00 [67], potassium, 187 [68] calcium, 109 [69] and available phosphorous, 5.6 [70] (all values of the nutrients were in mgkg^−1^ of dry soil). The soil electrical conductivity (ECe) was 2.1 dSm^−1^ and soil pH was 8.1 [71]. Electrical conductance (ECe), pH and inorganic nutrients of the soil saturation extract were appraised following Jackson [72].

### 2.6. Data Collection 

After 15 days of foliar spray, data for different growth, physiological and biochemical attributes was collected in morning. For the estimation of biochemical analysis, fresh leaf material third from top was collected and stored at −80 °C. 

### 2.7. Data Collection for Morphological and Growth Attributes

Two plants per replicate from each treatment were uprooted in the morning for the determination of root and shoot fresh biomass. Roots were then washed and blotted with paper to absorb the excess water from roots surface. Plants were then separated into roots and shoots. These samples were then weighed for the estimation of fresh weights of roots and shoots using an electric balance. These samples were then oven-dried for 72 h at 65 °C for the determination of dry mass of roots and shoots. The same plants were also used for the estimation of morphological attributes such as number of leaves and shoot length per plant. The oven-dried shoot samples were also used for the estimation of nutrients.

### 2.8. Estimation of Plant Water Relations

Leaf water potential (Ψ_w_) was measured in the morning from 6–8 a.m. A fully mature third leaf from top (two leaves per replicate) was excised and used for the measurement of the leaf Ψ_w_ using a pressure chamber, Model 615D, PMS Instrument Company, 1725 Geary Street SE Albany, OR 97322, USA. After measuring the Ψ_w_ the same leaf was placed in a freezer at −20 °C for at least 8 days. The frozen leaf was then thawed, and the cell sap was extracted. Then, the cell sap (10 µL) was directly used for the determination of leaf osmotic potential (Ψ_s_) using an osmometer (Wescor 5500, Artisan Technology Group 101 Mercury Drive Champaign, IL 61822). The leaf turgor pressure (Ψ_p_) was determined using the following formula:

Ψ_p_ = Ψ_w_ − Ψ_s_

### 2.9. Estimation of Leaf Relative Water Content (LRWC)

LRWC of fully expanded fresh leaves (third from the top and two leaves per replicate) were excised and leaf fresh weights were determined. The leaves were then numbered using a permanent marker and placed in dH_2_O (for 4 h) and then the turgid weight of leaves was measured after removing the excess water from surface leaves using a blotting paper. The same leaves were then oven-dried at 70 °C for 48 h and the dry weights of leaves were then measured. LRWC was measured using the following formula.

$$\mathrm{LRWC}\left(\%\right)=\left[\frac{\left(\mathrm{Leaf}\;\mathrm{fresh}\;\mathrm{weight}-\mathrm{Leaf}\;\mathrm{dry}\;\mathrm{weight}\right)}{\mathrm{Leaf}\;\mathrm{turgid}\;\mathrm{weight}-\mathrm{Leaf}\;\mathrm{dry}\;\mathrm{weight}}\right]\times 100$$

### 2.10. Estimation of Leaf Gas Exchange Attributes

Gas exchange parameters including sub-stomatal CO_2_ concentration (*C_i_*), stomatal conductance (*g_s_*), transpiration rate (*E*) and net CO_2_ assimilation rate *(A*) were studied using a portable infra-red gas analyzer (IRGA) LCA-4 ADC (Analytical Development Company, Hoddesdon, England). Fully developed third leaf from the top was used for the estimation of different gas exchange attributes. The estimation was performed from 9 a.m. to 12 p.m. The average light intensity during the estimation of parameters was 4.68 kWh/m^2^/d to 5.54 kWh/m^2^/d.

### 2.11. Estimation of Leaf Photosynthetic Pigments

Leaf total chlorophyll (T. Chl.), chlorophyll *b* (Chl. *b*), chlorophyll *a* (Chl. *a*) and carotenoid contents were measured using the method described by Arnon [73]. Briefly, fresh leaf material (0.1 g) two from each replicate was chopped into small pieces (1 cm) and leaf photosynthetic pigments were extracted using 10 mL of 80% acetone. The leaf material in acetone was then placed overnight and centrifuged at 10,000× *g* for 15 min. The absorbance of the supernatant was then read at 663, 645 and 480 nm. Chl. *a* and Chl. *b* were calculated using the following formula.

T. Chl = [20.2 (DA 645) − 8.02 (OD 663)] × V/1000 × W

Chl. *b* = [22.9 (OD 645) − 4.68 (OD 663)] × V/1000 × W

Chl. *a* = [12.7 (OD 663) − 2.69 (OD 645)] × V/1000 × W

V = volume of the extract (mL)
W = weight of the fresh leaf tissue (g)

However, for the estimation of leaf carotenoid contents, the formula given by Kirk and Allen [74] was used.

Carotenoids (mg mL^−1^) = A car/Em 100% × 100

A Car (carotenoid) = (OD 480) + 0.114 (OD 663) − 0.638(OD 645)

Em (Emission) = Em 100% = 2500

### 2.12. Estimation of Enzymatic and Non-Enzymatic Antioxidant 

#### 2.12.1. Extraction of Enzymatic Antioxidants and Total Soluble Protein

Fresh leaf sample (0.5 g) was homogenized using a pestle and mortar in 10 mL of 50 mM pre-chilled phosphate buffer with pH 7.8. The homogenate was then centrifuged at 10,000× *g* for 15 min at 4 °C. The supernatant so collected was used for enzyme assays and total soluble protein. 

#### 2.12.2. Determination of Superoxide Dismutase (SOD)

Activity of SOD was measured using the method as described by Giannopolitis and Ries [75]. For the preparation of reaction mixture, 50 µL of enzyme extract was added to a prepared mixture (1 mL) consisting of 50 μM NBT (NBT prepared solution in formamide), 13 mM methionine, 1.3 μM riboflavin, 75 nM EDTA and 50 mM phosphate buffer (pH 7.8). The reaction mixture was placed under a 20 V bulb for 15 min in a chamber internally coated with aluminum foil. Riboflavin was added to the reaction mixture before placing the mixture under the light source. A blank sample was also prepared each time without adding any extract. The absorbance (Abs) of the reaction mixture was measured to be 560 nm using a spectrophotometer. 

#### 2.12.3. Determination of Peroxidase (POD), Catalase (CAT) and Ascorbic Peroxidase (APX)

CAT and POD activities were measured using the method as given by Chance and Maehly [76]. The reaction mixture (3 mL) contained 50 mM phosphate buffer (pH 7.8), 59 mM H_2_O_2_, and 0.1 mL enzyme extract. The enzyme extract (100 µL) was mixed in last to start the reaction. The change in Abs was recorded at 240 nm for 120 s at intervals of 20 s. For the estimation of POD activity, the reaction mixture was prepared using 0.1 mL enzyme extract, 40 mM H_2_O_2_, 20 mM guaiacol and 50 mM phosphate buffer (pH 7.0). The change in the absorbance was measured at 470 nm for 120 s at intervals of 20 s. The APX activity was measured following the method described by Asada and Takahashi [77]. The decrease in the absorbance was measured at 290 nm for 120 s with intervals of 20 s. The reaction mixture was prepared by adding 2.1 mL phosphate buffer, 300 µL ascorbate (0.5 mM), 300 µL H_2_O_2_ and 300 µL of enzyme sample. 

#### 2.12.4. Estimation of Leaf Total Phenolic Content (TPC)

Leaf TPC was measured following the method described by Julkenen-Titto [78]. For this 0.05 g of fresh leaf sample was ground in acetone (80%) and then centrifuged at 10,000× *g* for 20 min. One hundred micro-liters of the supernatant was reacted with 1 mL of Folin–Ciocalteau’s phenol reagent and 2 mL of distilled water was added. Then 5 mL of Na_2_CO_3_ (20%) was mixed with the solution and the volume of the mixture was made up to 10 mL using distilled water. The Abs of the triturate was read at 750 nm. The TPC quantification was made by using an absorbance curve prepared from known standards prepared from catechin.

#### 2.12.5. Estimation of Leaf Ascorbic Acid (AsA) Content

Leaf AsA content was measured using the method described by Mukherjee and Choudhuri [79]. Fresh leaf material (0.25 g) was homogenized in 5 mL of 6% TCA solution. The homogenate was then centrifuged at 10,000× *g* for 20 min. In 4 mL of leaf extract, 2 mL of acidic dinitrophenyl hydrazine solution (2%) was added. A drop of 10% thiourea (prepared in 70% ethanol) was also added to mixture. The prepared mixture was heated in a water bath for 20 min at 95 °C. After cooling, 5 mL of H_2_SO_4_ (80%) was reacted with the mixture and the Abs of the finally prepared colored material was read at 530 nm using a UV visible spectrophotometer. 

#### 2.12.6. Estimation of MDA Content

For the estimation of the MDA levels in the samples, Heath and Packer’s [80] method was used. Briefly, in 10 mL of 0.1% trichloroacetic acid (TCA) solution, 1 g of sample was ground well and centrifuged for 15 min at 5000× *g*. Thiobarbituric acid (TBA) (0.5%) was prepared in 20% TCA and 0.5 mL was mixed into the supernatant. Then the mixture was heated at 95 °C for 50 min in a shaking water bath. After heating well, the tubes containing solution were directly placed in chilled water to terminate the reaction. The Abs of the final prepared mixture was read at 600 nm and 532 nm and the MDA levels were calculated using the following formula.

MDA level (nmol) = Δ (A 532 nm − A 600 nm)/1.56 × 10^5^

The absorption coefficient for calculating MDA is 1.56 mmol^−1^cm^−1^.

#### 2.12.7. Determination of H_2_O_2_ Content

The content of H_2_O_2_ in leaf samples was measured using the method given by Jena and Choudhuri [81]. Fresh leaf sample (0.5 g) was homogenized in 10 mL of TCA and then centrifuged at 10,000× *g* for 15 min. The obtained supernatant (0.5 mL) was added to a solution containing 1 mL KI and 0.5 mL phosphate buffer, and the absorbance was measured at 390 nm using spectrophotometer. The method was carried out in a minimal light condition.

### 2.13. Quantification of Leaf Different Amino Acids

#### 2.13.1. Quantification of Leaf Glycine Betain (GB) Content in Leaf

Leaf GB concentration was estimated using the method of Grieve and Grattan [82]. Fresh leaf samples (0.1 g) were chopped (0.1 cm) and extracted in ddH_2_O by shaking overnight. The samples were then filtered by Watman No.1 filter paper. To 1 mL of the filtrate, 1 mL of HCL (2 N) was added. Then the 0.5 mL of reaction mixture was mixed with KI solution (0.2 mL). Then 20 mL of dichloromethane (chilled) and 2 mL ddH_2_O (chilled) were added to the reaction mixture followed by continuous shaking along under flowing air. After the mixture was settled, the lower layer was taken, and the absorbance was measured at 365 nm using a spectrophotometer. Leaf GB content was quantified from a standard curve was prepared from known standards (5–25 ppm) following the same procedure. 

#### 2.13.2. Quantification of Leaf Proline Content

The measurement of proline in fresh leaf samples was performed using the method given by Bates et al. [83]. For this, 0.1 g fresh leaf sample was ground in 5 mL of 3% solution of sulfosalicylic acid followed by filtration. Then phosphoric acid (6 M) was added to 100 µL of the filtrate. The prepared solution was then mixed with 2 mL of each of glacial acetic acid and ninhydrin followed by heating at 95 °C in a water bath for 1 h. After heating, the tubes were immediately placed in chilled water for cooling and 4 mL of toluene was added with shaken. The absorbance of the final prepared mixture was measured at 520 nm using a spectrophotometer. The content of proline was measured by using a standard curve prepared from pure standards (5–25 ppm) following the same procedure.

Proline μmol g^−1^ Fw = mL of toluene/115 g × μg proline mL^−1^)/sample (g)

#### 2.13.3. Determination of Leaf Aspartate (Asp) Concentration in Leaf Samples

Leaf Asp concentration was estimated using the method described by Pfleiderer et al. [84]. The reaction mixture contained 0.1 mL dinitrophenylhydrazine (DPNH), 0.02 mL of malic dehydrogenase (600 units), 2.7 mL of phosphate buffer and 0.03 mL of transaminase (30 units) in 0.1 mL of leaf extract. The absorbance of the mixture was read at 340 nm. Then 0.05 mL of α-ketoglutarate was added to reaction mixture and the decrease in absorbance was recorded for 15 min. Aspartic acid content was quantified using the following formula

C = E × 133/6.22 × 10^6^ (g aspartic acid/mL)

6.22 × 10^6^ is the molar extinction coefficient of DPNH at 340 nm, 133 = molecular weight of aspartic acid, where E = extra plotted extinction decrease.

#### 2.13.4. Estimation of Leaf Glutamate (Glu) Concentration in Fresh Leaves

Briefly, the reaction mixture was prepared by adding diaphorase (0.14 i.u./mL), NAD (0.38 mM), INT (0.068 mM), 500 μL in a buffer-extracted sample of triethanolamine (57 mM), potassium phosphate (14 mM, pH 8.6) and GDH (14 i.u./mL). The basic mechanism behind the reaction is the formation of 2-oxoglutarate by the oxidative deamination in the presence of glutamate dehydrogenase and NAD. The formation of iodonitrotetrazolium chloride (INT) with diaphorase takes place through an oxidative process. The absorbance of the reaction mixture was read at 492 nm [85].

#### 2.13.5. Estimation of Lysine (Lys) and Methionine (Meth) Concentration in Leaves

The method described by Losak et al. [86] was used for the estimation Meth and Lys contents. In 10 mL of HCL (0.1%) fresh leaf samples (1 g) were ground by mortar and pestle. The samples were centrifuged at 10,000× *g* and were divided into two parts. For the estimation of Lys, ninhydrin solution, and 50% glycerol were added into one-part phosphate buffer (50 mM) and heated at 95 °C. The absorbance of the final prepared mixture was measured at 570 nm using a spectrophotometer. For the estimation of Meth in remaining part of leaf extract, NaOH (5 N), sodium nitroferricyanide dihydrate (0.1%), glycine dihydrate (50%), and HCl (1:1) were added and the absorbance was measured at 510 nm. 

### 2.14. Estimation of Yield Attributes

Two plants per replicate were harvested at maturity for the determination of different yield attributes. Cobs were placed in sunlight after separating them from the plants at physiological maturity. The grains were separated from the cobs manually. The yield parameters, such as 100-grain weight, grain yield/plant and total number of grains/cob were estimated. 

### 2.15. Estimation of Mineral Nutrients

For the estimation of different mineral nutrients, leaf dry samples were ground to a fine powder and digested with H_2_SO_4_ as per the method given by Wolf [87]. Different cations (Ca^2+^, K^+^, Mg^2+^, Fe^2+^ and Zn^2+^) were quantified using the atomic absorption spectrophotometer (AAS) (Hitachi. Model 7JO-8024, Tokyo, Japan). Phosphorous and nitrogen in the digested samples were estimated using the spectrophotometric and micro Kjeldhal method as described by Bremner and Keeney [88].

### 2.16. Statistical Analysis

The obtained data for the studied attributes was analyzed statistically using the Co-STAT Windows version 6.3 (developed by Cohort Software Berkley, CA, USA) to find out the significant differences among the treatments. The least significant difference (LSD) test at 5% level of significance was used to find out significant differences among mean values. Correlations and principal component analyses (PCA) of the studied attributes were computed using XLSTAT software. Significance among the generated values against each attribute for the correlations was determined using the Spearman’s correlation table.

## 3. Results 

### 3.1. Growth and Morphological Parameters

**Figure 1:**
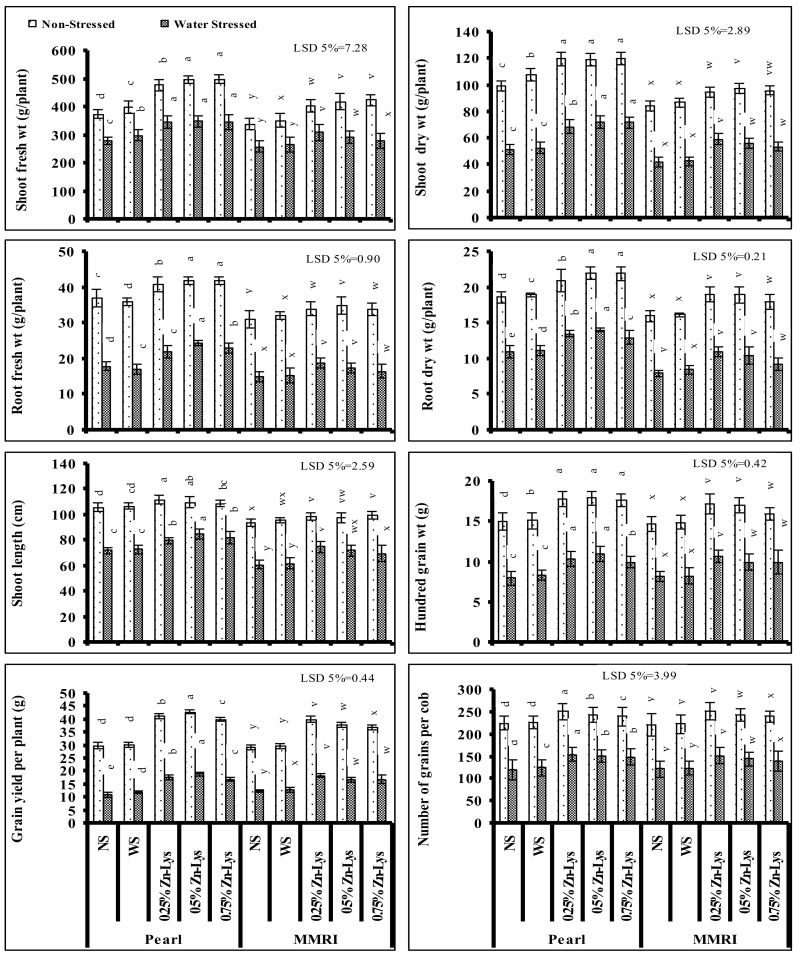
Growth and yield parameters of two maize cultivars foliar supplied with different levels of Zn-Lys when grown under different irrigation levels (Mean ± SE; *n* = 4). NS = no spray; WS = water spray; Zn-Lys = Zn-lysine. [Bars with different letters differ significantly; a, b, c… and x, y, z… refer to cultivars Peal and MMRI, respectively, under both water regimes].

Shoot fresh and dry weights of both maize genotypes were adversely affected when grown under reduced irrigation. Fertigation of varying regimes of Zn-Lys as foliar spray significantly reduced the deleterious impact of deficit irrigation on these growth parameters. This improvement in shoot fresh and dry weights due to foliar-applied Zn-Lys was also found in maize plants of both genotypes grown under normal irrigation. However, the increase was maize cultivar- and Zn-Lys level-specific. In case of cv. Pearl, regarding shoot fresh weight, all three levels of Zn-Lys were equally effective in ameliorating the negative impacts of water shortage. Regarding the cv. MMRI, the 0.25% level of Zn-Lys was found to be superior in comparison with other two levels. However, under normal irrigation conditions, the Zn-Lys-induced increase in shoot fresh weight of both maize cultivars was higher in maize plants fertigated with 0.5 and 0.75% levels. Regarding shoot dry weight in case of cv. Pearl, the 0.5 and 0.75% levels of Zn-Lys were found to be better, and the 0.25% level was found better for cv. MMRI in reducing the negative impacts of deficit irrigation. However, under normal irrigation, plants fertigation with all levels of Zn-Lys showed equal improvement in shoot dry weight in cv. Pearl, but the 0.5% level of Zn-Lys was the most effective in cv. MMRI. Zn-Lys-applied. This improvement in shoot fresh and dry biomasses was greater in maize plants of both cultivars grown under deficit irrigation in comparison with the normally irrigated plants. It shows the stress ameliorative role of foliar-applied Zn-Lys in water-stressed maize plants. However, the stress ameliorative effect was Zn-Lys dose- and cultivar specific. Regarding the shoot dry weight, the maximum increase was found in the 0.5% (27.8 and 16.66% increase) and 0.25% (29.47 and 13.26% increase) Zn-Lys in cv. Pearl and MMRI under deficit irrigation and normal irrigation conditions, respectively (Figure 1). 

Fresh and dry biomass of roots were also significantly reduced in both maize genotypes under deficit irrigation. Fertigation with all levels of Zn-Lys significantly reduced the negative impacts of water shortage on fresh and dry biomass of roots. In the case of cv. Pearl, 0.5 and 0.75% levels of Zn-Lys were found to be equally effective in improving the root fresh weight in normally irrigated plants, while under water deficit conditions, the 0.5% level of Zn-Lys was the most effective, followed by the 0.75% level. Regarding cv. MMRI, 0.5 and 0.25% levels of Zn-Lys were the most effective under deficit irrigation conditions and the 0.5% level was most effective in normally irrigated plants. Regarding the root dry weight in cv. Pearl, under non-stress conditions 0.5% and 0.75% levels of Zn-Lys were more effective, while under water deficit conditions, the 0.5% level of Zn-Lys was superior. Regarding cv. MMRI, the 0.25 and 0.5% levels were most effective in improving root dry weight in deficit irrigation as well as in well irrigated conditions. 

Shoot length (SL) of both maize cultivars was also significantly decreased due to deficient irrigation. Fertigation with Zn-Lys as a foliar spray ameliorated the negative impacts of deficient irrigation on SL of both maize genotypes. Zn-Lys-induced increase in SL was also found in maize plants grown under normal irrigation. However, the ameliorative effect was maize cultivar- and Zn-Lys level-specific. Under deficit irrigation, in the case of cv. Pearl, the 0.5% level of Zn-Lys was found more effective, while for cv. MMRI, the 0.25% level of Zn-Lys was the best. However, under non-stressed conditions, the 0.25% level of Zn-Lys was superior in improving the SL in cv. Pearl, while in case of cv. MMRI, all regimes of Zn-Lys showed a similar response in improving the plant SL (Figure 1). 

### 3.2. Yield Attributes

Significant reductions in the studied yield parameters including the hundred-grain weight, number of grains per cob and grain yield per plant were found under deficit irrigation in both maize genotypes. Fertigation with all regimes of Zn-Lys decreased the negative impacts of reduced irrigation on all yield parameters. Improvements in yield parameters were also found in plants grown under normal irrigated conditions due to foliar spray with different levels of Zn-Lys. This improvement in yield attributes was greater in maize plants of both cultivars grown under deficit irrigation in comparison with normally irrigated plants. This shows the stress ameliorative role of foliar-applied Zn-Lys in water-stressed maize plants. However, the stress ameliorative effect was Zn-Lys dose- and cultivar specific. Regarding the grain yield per plant, the maximum increase was found at 0.5% (42 and 30% increase) and 0.25% (31.69 and 26.35% increase) levels of Zn-Lys in cv. Pearl and MMRI under deficit irrigation and normally irrigated conditions, respectively. Regarding the improvements in number of grains per cob, the 0.25% level of Zn-Lys was found to be more effective for both maize genotypes under both irrigation regimes. Regarding the hundred-grain weight, in cv. Pearl under water deficit conditions, improvement was found in plants fertigated with 0.25 and 0.5% levels of Zn-Lys, but in cv. MMRI, the 0.25% level of Zn-Lys was comparatively more effective. However, in the case of normally irrigated cv. Pearl, all regimes of Zn-Lys showed a similar response in improving the hundred-grain weight, but regarding cv. MMRI, 0.25% and 0.5% levels of Zn-Lys were the most effective (Figure 1). 

### 3.3. Gas Exchange Attributes

**Figure 2:**
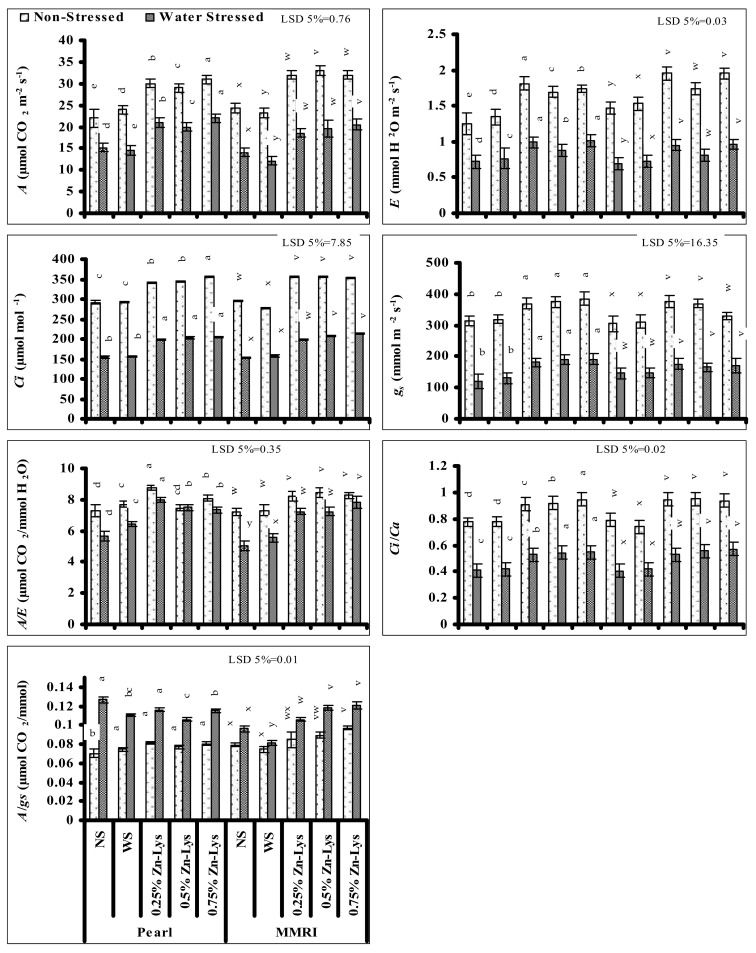
Gas exchange attributes of two maize cultivars foliar-supplied with different levels of Zn-Lys when grown under different irrigation levels (Mean ± SE; *n* = 4). NS = no spray; WS = water spray; Zn-Lys = Zn-lysine; *E* = leaf transpiration rate; *A* = leaf net photosynthetic rate; *g_s_* = leaf stomatal conductance; *A*/*E* = leaf water use efficiency; *C_i_* = intrinsic CO_2_ concentration; *A*/*g_s_* = leaf intrinsic water use efficiency; *C_i_*/*C_a_* = ratio of leaf intrinsic CO_2_ concentration to ambient CO_2_ concentration. [Bars with different letters differ significantly; a, b, c… and x, y, z… refer to cultivars Peal and MMRI, respectively under both water regimes].

Leaf transpiration rate (*E*) and net photosynthetic rate (*A*) reduced significantly in both maize genotypes when grown under water deficit stress. Foliar-application of Zn-Lys significantly reduced the negative impacts of water deficit stress on both photosynthetic parameters in both maize genotypes. The Zn-Lys-induced improvements in *A* and *E* were also recorded in normally irrigated plants of both maize cultivars. However, the effect was maize cultivar- and Zn-Lys dose specific. Regarding *A*, this increase was slightly greater in maize plants of cv. Pearl supplied with the 0.75% level of Zn-Lys under both water regimes. While in cv. MMRI, 0.75 and 0.5% levels of Zn-Lys were comparatively better in water deficit and normal irrigated plants, respectively (Figure 2). Regarding *E*, the 0.25 and 0.5% levels of Zn-Lys were found to be more effective in ameliorating the negative impacts of limited irrigation in both maize genotypes. However, under normal irrigation, in the case of cv. Pearl the 0.25% level, and in cv. MMRI the 0.25 and 0.75% levels were the most effective in improving *E* (Figure 2). 

Significant adverse impacts of limited irrigation were also recorded on other gas exchange attributes such as *C_i_*, *g_s_* and *C_i_*/*C_a_* in both maize genotypes. Foliar-applied Zn-Lys was found to be effective in reducing the negative effects of limited irrigation on *C_i_*, *g_s_* and *C_i_*/*C_a_* in both maize cultivars but the effectiveness was maize cultivar- and Zn-Lys dose specific. Zn-Lys-induced improvements in these attributes were found under normal irrigation in both genotypes. During water stress, regarding improvement in *C_i_* in cv. Pearl, all foliar-applied Zn-Lys levels were found to be equally effective, and the 0.75% level was found to be the most effective one under normal irrigation. However, in case of cv. MMRI in water-limited plants, 0.5 and 0.75% regimes of Zn-Lys were found to be superior, however in normally irrigated plants, all the levels of Zn–Lys showed the same improvement in *C_i_*. Regarding the *g_s_*, all three levels of Zn-Lys showed equal effectiveness in both maize genotypes both under water deficit stress and non-stressed conditions, except in cv. MMRI under normal irrigation, where the 0.75% level of Zn-Lys was not effective in improving the gs. Improvements in *C_i_*/*C_a_* were also maize cultivar- and Zn-Lys dose specific. In cv. Pearl, the 0.75% level of Zn-Lys was found to be superior under both water regimes. In the case of cv. MMRI, all levels of Zn-Lys showed similar improvement effects in *C_i_*/*C_a_* in well-irrigated plants, but during limited irrigation the 0.5 and 0.75% regimes of Zn-Lys were superior (Figure 2). 

Significant reduction in water use efficiency (*A*/*E*) due to limited water supply was recorded in both maize genotypes and a larger decrease was recorded in cv. MMRI. Foliar spray of Zn-Lys significantly improved the *A*/*E* of both maize genotypes under both water deficit stress and normal irrigation, but the increase was Zn-Lys level- and maize cultivar specific. In water deficit-stressed plants, all levels of Zn-Lys significantly improved the *A*/*E* in both maize cultivars, but the 0.25% and 0.75% levels were the most effective ones in cv. Pearl and MMRI, respectively. However, under non-stressed conditions, Zn-Lys-induced improvement in *A*/*E* was recorded only in maize plants supplied with the 0.25% level in cv. Pearl, while in cv. MMRI, all levels of Zn-Lys gave equal response in improving leaf *A*/*E*. Furthermore, the improvement in *A*/*E* due to Zn-Lys foliar spray in both maize cultivars was found in the plants with limited water supply in comparison with normally irrigated plants (Figure 2).

Significant increases in leaf intrinsic water use efficiency (*A*/*g_s_*) were recorded in both maize cultivars when grown under limited water supply with the greater improvement being found in cv. Pearl. Foliar-applied Zn-Lys significantly affected the *A*/*g_s_* in both maize genotypes under both irrigation regimes; however, the extent of increase or decrease was maize genotype specific. In cv. Pearl, all levels of Zn-Lys significantly reduced the *A*/*g_s_* but only under water deficit conditions, and comparatively less reduction was recorded at the 0.5% level of Zn-Lys. However, in normally irrigated plants, *A*/*g_s_* increased at all levels of Zn-Lys. Regarding cv. MMRI, an improvement in *A*/*g_s_* was found both under limited irrigation and normal irrigation conditions and the largest increase was found with the 0.75% level of Zn-Lys (Figure 2).

### 3.4. Plant–Water Relations

**Figure 3:**
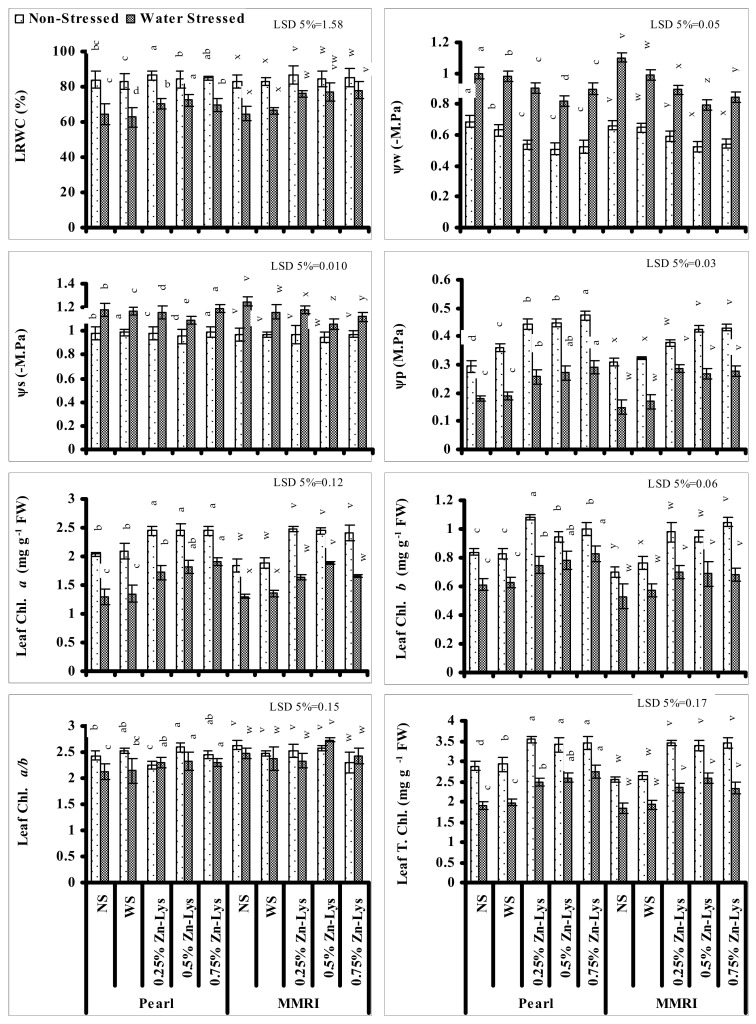
Photosynthetic pigments and water relations of two maize cultivars foliar supplied with varying levels of Zn-Lys when grown under different irrigation levels (Mean ± SE; *n* = 4). WS = water spray; NS = no spray; Zn-Lys = Zn-lysine; Ψ_p_ = leaf turgor potential; Ψ_s_ = leaf osmotic potential; Ψ_w_ = leaf water potential; LRWC = leaf relative water content; Chl. *a* = leaf chlorophyll a content; Chl. *b* = leaf chlorophyll *b* content; Chl. *a*/*b* = ratio of leaf chlorophyll *a* to leaf chlorophyll *b*; T. Chl. = leaf total chlorophyll. Bars with the same letter do not differ significantly; a, b, c… and x, y, z… refer to cultivars Peal and MMRI, respectively, under both water regimes.

Significant decreases in leaf relative water content (LRWC) were recorded in both maize genotypes under reduced irrigation. Foliar spray with different regimes of Zn-Lys significantly decreased the negative impact of reduced irrigation on LRWC but the extent of amelioration was maize cultivar- and Zn-Lys level specific. In cv. Pearl, 0.50% and 0.25% levels were the most effective in improving LRWC in water deficit and normal irrigation, respectively. However, in case of cv. MMRI, 0.75 and 0.25% levels of Zn-Lys were found to be the most effective under limited irrigation and normal irrigation, respectively (Figure 3). 

Similarly, significant reductions in Ψ_w_ and Ψ_s_ were also recorded under limited water supply in both maize genotypes. Foliar-applied regimes of Zn-Lys improved the Ψ_w_ and Ψ_s_ significantly in both maize cultivars. The maximum improvement in leaf Ψ_w_ and Ψ_s_ were recorded in maize plants foliar-applied with the 0.50% level of Zn-Lys in both maize cultivars when grown in water deficit stress. Zn-Lys induced improvements in Ψ_w_ and Ψ_s_ were also recorded in both maize cultivars grown under normal irrigation, but the amelioration was cultivar specific. Regarding Ψ_w_, Zn-Lys-induced improvement was only in cv. MMRI at 0.5 and 0.75% levels. However, regarding Ψ_s_, the maximum improvement in both cultivars was with the 0.5% level of Zn-Lys (Figure 3).

Significant reductions in leaf Ψ_p_ were also found in both maize genotypes when grown under reduced water supply and a comparatively larger decrease was recorded in cv. MMRI compared with cv. Pearl. Significant increases were recorded in leaf Ψ_p_ in both maize cultivars under reduced irrigation as well as under normal irrigation, but the improvement was Zn-Lys level- and cultivar specific. Under water deficit conditions, the 0.75% level was found to be most effective in cv. Pearl under both water regimes, in improving the leaf Ψ_p_. However, in case of cv. MMRI when grown under normal irrigation, 0.5 and 0.75% of Zn-Lys were the most effective, but under reduced irrigation, all Zn-Lys levels showed similar increases (Figure 3).

### 3.5. Photosynthetic Pigments

Leaf T. Chl., Chl. *a* and *b* contents significantly decreased in both maize genotypes when grown under reduced irrigation. All levels of foliar-applied Zn-Lys significantly increased the contents of leaf T. Chl., Chl. *a*, and *b* of both maize cultivars under both water regimes. However, the most effective level was maize cultivar- and chlorophyll type specific. Regarding Chl. *a* under reduced irrigation, 0.75 and 0.5% levels of foliar-applied Zn-Lys were the most effective in improving the Chl. *a*, in cv. Pearl and MMRI, respectively, but under normal irrigation, all levels of Zn-Lys showed similar increasing effects in improving the leaf Chl. *a*. Regarding Chl. *b*, 0.25 and 0.75% levels of Zn-Lys were most effective under normal irrigation and water deficit, respectively in cv. Pearl. However, in cv. MMRI all Zn-Lys levels showed a similar effect in increasing Chl. *b* content under water deficit stress, but under normal irrigation, the 0.75% level was most effective. Regarding the leaf T. Chl., under water stress the 0.75% level of Zn-Lys in cv. Pearl, and all three levels of Zn-Lys in cv. MMRI, were found to be effective, but under normal irrigation all Zn-Lys levels showed similar response in improving the leaf T. Chl. content in both maize cultivars (Figure 3).

Reduced irrigation also significantly reduced the leaf Chl. *a*/*b* in both maize cultivars. Foliar-supplied Zn-Lys in both maize cultivars significantly affected the leaf Chl. *a*/*b* under both water regimes. However, the effect was maize cultivar- and Zn-Lys level specific. Under water deficit stress, regarding cv. Pearl, all levels of Zn-Lys showed similar increases in leaf Chl. *a*/*b*, but regarding cv. MMRI, the improvement in Chl. *a*/*b* was found only in water-stressed plants foliar-supplied with 0.5% level of Zn-Lys (Figure 3). However, under normal irrigation, Zn-Lys-induced increase or decrease was not specific. 

### 3.6. MDA and H_2_O_2_ Contents

**Figure 4:**
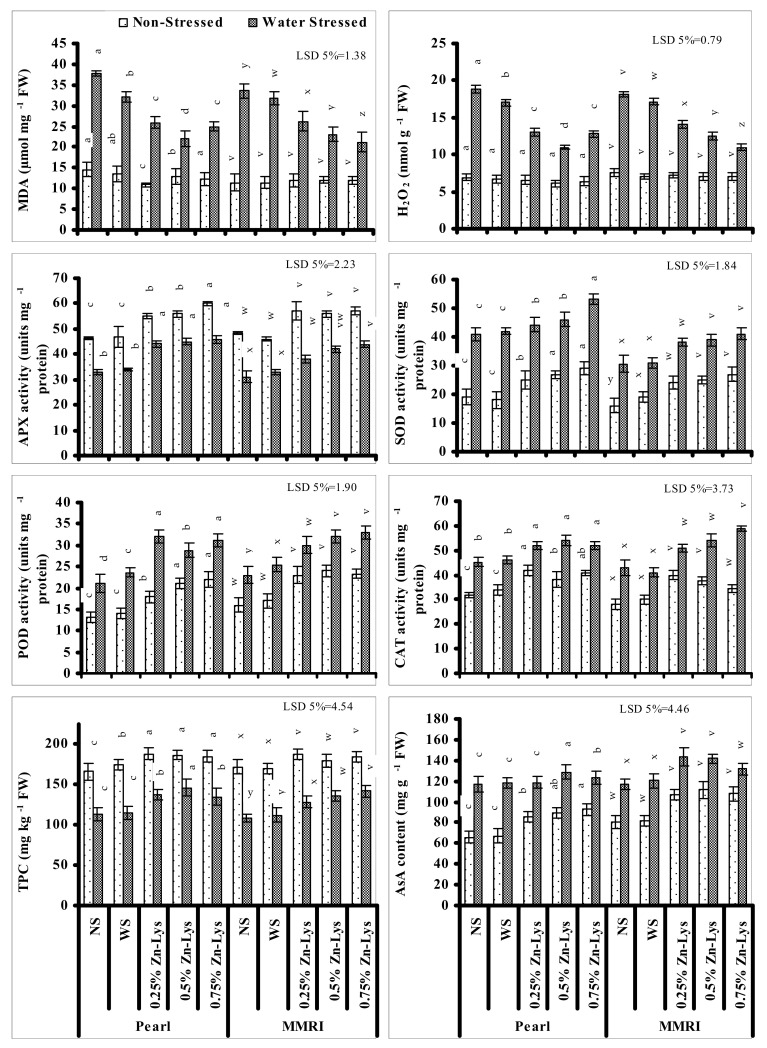
Leaf MDA, H_2_O_2_, leaf AsA, leaf TPC, activities of SOD, POD, APX, and CAT of two maize cultivars foliar-supplied with different levels of Zn-Lys when grown under different irrigation levels (Mean ± SE; *n* = 4). NS = no spray; WS = water spray; Zn-Lys = Zn-lysine; MDA = leaf malondialdehyde; leaf AsA = leaf ascorbic acid content; leaf TPC = leaf total phenolic content; APX = ascorbate peroxidase; POD = peroxidase; SOD = superoxide dismutase; CAT = catalase. Bars with different letters differ significantly; a, b, c… and x, y, z… refer to cultivars Peal and MMRI, respectively, under both water regimes.

MDA and H_2_O_2_ contents increased significantly under limited water supply in both maize cultivars, showing the increased damage due to oxidative stress. Fertigation with all regimes of Zn-Lys as a foliar spray significantly decreased the negative impact of limited water supply regarding reduced levels of H_2_O_2_ and MDA in both studied maize genotypes. Among all studied Zn-Lys levels, the maximum reduction in MDA and H_2_O_2_ in cultivars Pearl and MMRI was found at 0.5% and 0.75% levels, respectively (Figure 4).

### 3.7. Enzymatic and Non-Enzymatic Antioxidants

Leaf APX activity decreased significantly in both maize genotypes and a comparatively greater decrease was found in cv. MMRI than cv. Pearl. Different levels of foliar-applied Zn-Lys significantly decreased the negative impacts of deficient irrigation on APX activity in both maize cultivars and the effect was maize cultivar- and Zn-Lys level specific. In cultivar Pearl, under deficient irrigation, fertigation with all regimes of Zn-Lys increased the APX activity equally, but under normal irrigation the 0.75% level was superior. While, in cv. MMRI under water deficit conditions, the 0.75% level was the most effective. Under normal irrigation, all regimes of Zn-Lys induced similar increases in APX activity (Figure 4). 

Leaf POD, CAT and SOD activities increased significantly when grown under deficit irrigation. Fertigation with Zn-Lys further increased the activities of SOD, POD and CAT in both maize genotypes both under deficient and normal irrigation conditions. The maximum improvement in SOD activity was found in plants of both maize cultivars fertigated with 0.75% Zn-Lys under both irrigation regimes. In the case of leaf POD activity in non-stressed plants of cv. Pearl, the 0.75 and 0.25% levels were found to be most effective, but under water deficit conditions 0.75% level was the most effective in increasing the leaf POD activity. However, in cv. MMRI under normal irrigation conditions, all levels of Zn-Lys showed a similar increasing trend, while under deficit irrigation the maximum increment in POD activity was found in plants fertigated with 0.5 and 0.75% levels. Regarding the effect of Zn-Lys on leaf CAT activity, all levels of Zn-Lys as foliar fertigation showed similar improvement in leaf CAT activity under deficit irrigation in cv. Pearl but in cv. MMRI, the 0.75% level was found to be most effective (Figure 4). However, in non-stressed plants of both maize cultivars, the 0.25% level of Zn-Lys was most effective.

Leaf total phenolic content decreased significantly under reduced irrigation in both maize genotypes. Foliar fertigation with different levels of Zn-Lys significantly reduced the negative impacts of deficit irrigation on leaf phenolic contents in both maize genotypes. However, the extent of amelioration was Zn-Lys level- and maize cultivar specific. Under deficit irrigation, the 0.5% and 0.75% levels of Zn-Lys were most effective in improving leaf total phenolic contents in cvs. Pearl and MMRI, respectively. However, under non-stressed conditions, all regimes of Zn-Lys showed similar increasing effects in leaf total phenolic content in cv. Pearl and the 0.25 and 0.75% levels were found most effective in cv. MMRI (Figure 4). 

Significantly increased Leaf AsA content was recorded in both maize genotypes when grown under deficit irrigation. Foliar-spray with different Zn-Lys regimes significantly increased the leaf AsA content in both maize genotypes under both irrigation regimes. Under deficit irrigation this increase was maximized at 0.50% and 0.25% levels in cv. Pearl and MMRI respectively. However, under normal irrigation all regimes of Zn-Lys in cv. MMRI, and 0.75% in cv. Pearl, were effective in increasing the leaf AsA content (Figure 4). 

### 3.8. Amino Acid Contents

Levels of studied all studied amino acids, Asp, Meth, Glu, proline and GB were significantly increased in both maize genotypes under deficit irrigation. However, the content of lysine was decreased significantly in both maize cultivars under deficit irrigation. Fertigation with different regimes of Zn-Lys as foliar spray significantly enhanced the levels of all studied amino acids under both normal irrigation and deficit irrigation in both maize genotypes. The maximum increase in these amino acids was recorded in plants foliar-supplied with the 0.75% level of Zn-Lys under both irrigation regimes in both maize cultivars, except for leaf Glu content in non-stressed plants of both maize genotypes, where the maximum increase was recorded in plants foliar-supplied with the 0.5% level of Zn-Lys (Table 1). In comparison, under water deficit stress, the percentage increase due to foliar-applied Zn-Lys in Glu (10.64 and 12.97%), proline (25.52 and 17.91%) and GB (27.05 and 26.67%) in cv. Pearl and MMRI, respectively, was greater compared with non-stressed conditions.

### 3.9. Shoot Nutrient Content 

Shoot nutrient contents, Ca, K, P, N and Fe of both maize genotypes also decreased significantly under deficit irrigation. However, the fertigation with Zn-Lys improved the nutrient content under both irrigation levels in both maize cultivars. The extent of the increase in shoot nutrient uptake was Zn-Lys level-specific under both irrigation regimes in both maize genotypes. It was found that the 0.5% level of Zn-Lys was more effective at increasing the Ca, K, P, N and Fe contents in cv. Pearl under deficit irrigation as well as under normal irrigation. In case of cv. MMRI, the 0.25% level of Zn-Lys was superior in improving the leaf N and Ca contents, 0.5% in improving Mg and Fe and 0.75% in improving leaf P content. Regarding leaf Zn content, the 0.75% level of Zn-Lys was found to be the most effective under both water regimes in both maize cultivars (Table 2).

### 3.10. Principle Component Aanalysis (PCA) and Correlations of Studied Attributes

**Figure 5:**
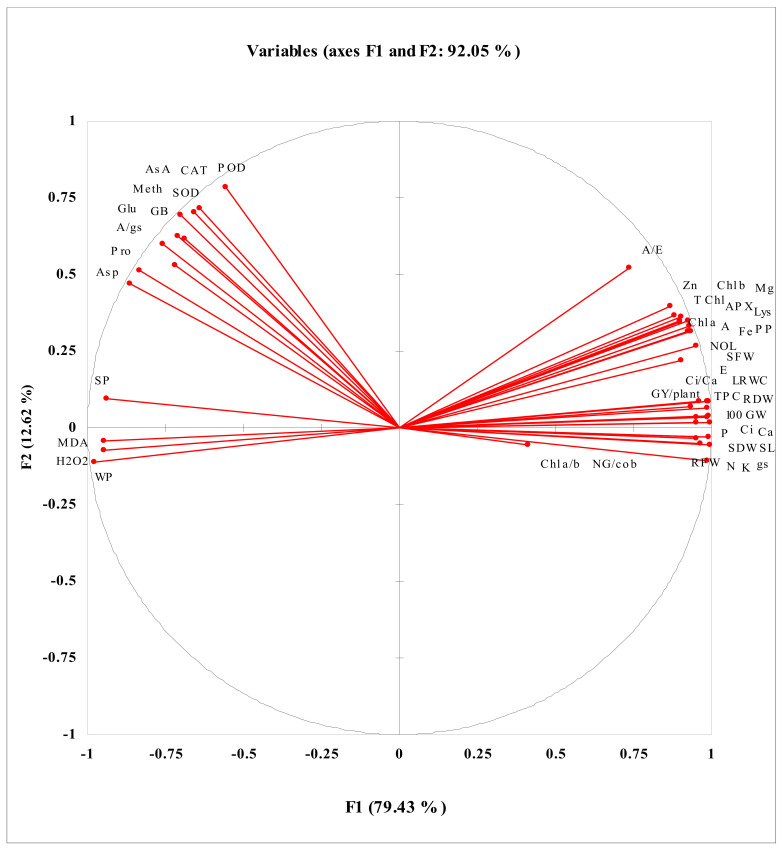
Principal component analysis (PCA) of different studied attributes.

Data for correlation and PCA analysis of studied attributes are presented in Figure 5 and Table 3. Strong positive correlation was recorded between different growth measures (SFW, SDW, RFW, RDW), yield (100 GW, GY/plant, NG/cob), leaf photosynthetic pigments (Chl. *a*, Chl. *b* and T Chl.), gas exchange attributes (*A*, *E*, *C_i_*, *g*_s_, *A*/*E*) mineral nutrients (K^+^, Ca^2+^, Mg^2+^, N, P), leaf TPC, leaf Zn and Lys content. On the other hand, activities of antioxidant enzymes (SOD, POD and CAT) and leaf ascorbic acid were closely positively correlated with amino acid contents (Meth, GB, Glu, Pro and Asp) and negatively correlated with MDA and H_2_O_2_. However, leaf MDA, H_2_O_2_ and WP were positively correlated with all other attributes. The parameters in circle I were negatively correlated with parameters in circles II and III (Figure 5; Table 3).

## 4. Discussion

Unavailability of fresh water for irrigation is considered a major obstacle in obtaining better crop yields. This situation is especially obvious in arid and semi-arid regions of the world [89]. It is expected that this situation will become more alarming in near future due to rapidly changing environmental conditions along with altered rainfall patterns [90,91] which have created aridity in some areas and flooding in others. Under such conditions, obtaining better plant growth and yield has become an important issue in crop plants. To overcome this problem, different strategies, including the use of chemicals, have gained interest among the researchers [6,7,11,92,93,94,95,96]. In present study, reduced irrigation significantly decreased the growth and yield of both maize genotypes and greater decreases were recorded in cv. MMRI. Water stress-induced reductions in biomass production and final seed yield are common phenomena in crop plants [97,98,99]. However, the extent of reductions in yield and biomass are crop species- and cultivar specific [100]. Reduction in grain yield is correlated well with reduced carbohydrate accumulation, assimilation and leaf photosynthetic efficiency [101]. The present study’s findings in relation to decrease in seed yield under reduced irrigation are similar to those reported in earlier studies [1,102]. 

The decrease in growth and grain yield is correlated well with plant–water relations and leaf photosynthetic efficiency along with nutrient assimilations [103,104] which are badly affected under limited water supply [104]. Similar perturbation in plant–water relations along with photosynthetic efficiency due to water stress are also found in the present study in both maize cultivars, being greater in the MMRI cultivar, showing its greater sensitivity to reduced irrigation. It was found by Hao et al. [105] that in maize, decrease in growth-related attributes correlated well with disturbance of plant–water relations along with plant photosynthetic activity. Exogenous use of Zn-Lys as foliar fertigation resulted in reducing the adverse impacts of deficient irrigation on plant–water relations (LRWC, Ψ_w_, Ψ_s_, Ψ_p_) and gas exchange attributes such as *A*, *E*, *C_i_*, *g_s_ A*/*E A*/*g_s_* and *C_i_*/*C_a_* in maize plants of both cultivars which resulted in greater plant biomass and yield, showing the positive influence of foliar-applied Zn-Lys in reducing the negative impacts of reduced irrigation by maintaining the better cellular water status [58,106,107,108,109], ultimately increased the final seed yield. Though the positive influences of Zn-Lys foliar spray were also recorded in non-stressed plants of both maize genotypes, these improvements were less than in water-stressed plants. Regarding the LRWC, the clear improvement due to foliar spray of Zn-Lys was noted only in the water deficit-stressed plants of both maize cultivars in comparison with normally irrigated plants (Figure 3). A similar trend was found in the case of leaf water use efficiency in both maize cultivars, strongly positively associated with the better LRWC. This clear improvement in LRWC and *A*/*E* under water deficit stress due to foliar spray with Zn-Lys seems responsible for the greater increase in yield and growth compared with non-stressed maize plants (Figure 1). In the present study this higher percentage increase in seed yield and growth in water-stressed maize plants due to Zn-Lys foliar spray compared with non-stressed plants shows the clear role of Zn-Lys in drought tolerance of maize cultivars by maintaining better plant–water relations. The higher values of *A/E* in Zn-lysine-treated maize plants shows the effective role of lysine. The findings are also similar to those of Rizwan et al. [106], who found that exogenous application of Zn-Lys significantly improved grain yield, in association with an increase in photosynthesis and enzyme activities in wheat plants grown in stressful conditions. Lysine directly catabolizes in the TCA cycle that maintains cell energy status for different metabolic activities. More biomass production results in the availability of new binding sites for the nutrient [110]. Therefore, the improvement in plant stress tolerance due to Zn-Lys application might also be due to the catabolism of lysine as a source of energy. This catabolism of Lys in the TCA cycle and improvement in Zn contents also could explain the improvement in plant nutrition that supported the greater biomass production and seed yield, not only under deficit irrigation but also non-stressed conditions due to foliar-applied Zn-Lys. Furthermore, Zn-Lys foliar application also improved the plant amino acid contents and nutrient uptake under both water regimes, which are positively linked with increased growth and yield. Similar findings have been reported earlier in field-grown sunflower [6] and ajwain plants [7], where foliar-applied Fe-Asp and Fe-Glu, respectively, significantly improved the biomass and seed yield both under deficit- and normal irrigation and greater improvements were reported when applied to water deficit-stressed plants in comparison with normally irrigated ones, indicating the water-stress tolerance-inducing role of micronutrient-chelated amino acids.

Moreover, cellular osmotic imbalance directly effects the plant–water relations and plant photosynthetic efficiency [111] leading to a decrease in growth and seed yield [112]. In the present study, water shortage seriously disturbed the plant–water relations, such as, LRWC, Ψ_s_, Ψ_p_ and Ψ_w_ along with plant photosynthetic efficiency regarding the gas exchange attributes, including leaf *A*, *E*, *g_s_*, Ci, *A*/*E* and *A*/*g_s_*, responsible for decreased biomass and grain yield. However, foliar-applied Zn-Lys significantly increased the plant–water relations and photosynthetic efficiency. This improvement due to Zn-Lys application in plant–water relations along with plant photosynthetic activity might be due to the significant combined role of Zn and lysine in maintaining better cellular water relations [112]. Zinc is directly involved in maintaining plant–water relations due to its role in the regulation of stomatal opening and closing [113], as well as by maintaining the membrane integrity [31,114]. Availability of Zn in the required quantity maintains the K^+^ influx in plant cells that is also necessary to regulate the stomatal functioning [115,116] and has a necessary role in proper photosynthetic efficiency as well as in maintaining the plant water content. This is clear in present study where Zn-Lys application maintained better LRWC and *A*/*E* in water deficit-stressed maize plants of both maize cultivars compared with non-stressed ones that shows its role in regulating the leaf *g_s_* especially under water deficit stress that is associated with better performance in growth and seed yield. Shemi et al. [117] reported that in water-stressed maize plants, a Zn foliar spray significantly improved the grain yield in association with improvements in cellular osmotic adjustments, photosynthetic activity, and water relations. In drought-stressed wheat, exogenously applied Zn increased the grain yield and its quality in association with improvements in water status and photosynthetic efficiency [118]. It was reported that in wheat, a Zn-Lys foliar spray increased significantly the seed yield under a stressful environment, a function of its significant role in improving the plant–water relations as well as plant gas exchange attributes [106]. Similarly, in water-stressed radish plants, an exogenous supply of Zn-Lys significantly increased root biomass, which was linked with the increased content of Zn and Lys [58]. 

Furthermore, it is well defined in numerous studies that leaf hydraulic conductance also plays a major role in plants’ tolerance to water deficit stress and has role in maintaining the plant–water relations, but it is plant species and cultivar specific [119,120]. Under limited soil water availability, tensions in plant and soil increased considerably, which reduced the capacity for water translocation, known as the leaf hydraulic conductance, calculated as the ratio of flow rate/Ψ_w_ will decline, resulting in a reduction in plant photosynthetic efficiency and plant growth, eventually leading to severe losses in crop yield [121,122]. Moreover, the Ψ_w_ of a plant at a given time under varied environmental conditions depends on *E* and plant/leaf hydraulic conductance [122]. Studies reveal that under adverse environmental conditions, the water potential of leaves experiences a significant decline in hydraulic conductance, but not in stems, supporting the well-declared hypothesis that leaves of plants act as a ‘safety-valve’ [123]. The capacity of leaves regarding the hydraulic conductance affects the functions of their anatomical differences in mesophyll cells and venation architectures [119,120]. So, in the present study the cultivar-specific differences in plant–water relations, especially the Ψ_w_ and LRWC, might be due to the differences in their hydraulic conductance and the function of leaf architectural properties [124].

In view of the mechanisms described, with an increase in *E*, any decline in hydraulic conductance will increase the decline in leaf Ψ_w_ and will cause closing of stomata in a passive mechanism due to turgidity loss in stomatal guard cells or due to active mechanisms with the loss of anions from guard cells [125]. Regarding the present study, under water stress, the decrease in leaf Ψ_w_ due to *E* in both maize cultivars can be correlated well with these studies and with a larger decrease in Ψ_w_ in genotype MMRI, which may show its relatively higher leaf hydraulic conductance compared with genotype Pearl. Such a mechanism is well described [120,124]. Plant species in which *g_s_* declines more rapidly under water deficit conditions maintain better leaf hydraulic conductance, show tolerance towards water deficit conditions, and enable better leaf Ψ_w_ [119]. This might also be the case in the present study where water-stressed plants of both maize genotypes maintained better *g_s_* due to the Zn-Lys foliar treatment, showing its role in better maintenance of leaf hydraulic conductance, which was comparatively greater in genotype Pearl. The better maintenance of *g_s_* in the present study is also linked with better LRWC and Ψ_w_, which might be the function of better maintenance of hydraulic conductance [119].

On the other hand, amino acids are reported to maintain cellular osmotic adjustment [58,103,106] that also regulates the accumulation of different osmoprotectants and osmoregulators. Foliar-supplied Zn-Lys significantly increased the levels of Zn and lysine, confirming its foliar absorption and translocation. Zn-Lys foliar application, which improved lysine content and increased the levels of GB, proline, Asp, Meth and Glu. Such accumulation has also been studied earlier in water-stressed sunflower and ajwain plants [7,35], when supplied foliary with micronutrient-chelated amino acids, such as Fe-Asp and Fe-Glu. They reported that micronutrient-chelated amino acids were found more effective in improving water stress tolerance than their respective chemical fertilizers by maintaining the plant’s water status through cellular osmotic adjustment. In the present findings, the increased accumulation of different amino acids due to foliar-applied Zn-Lys was also linked with improved cellular water relations. It is well known that amino acids have important roles in maintaining better cellular water relations through cellular osmotic adjustment [7,35]. Similarly in the present study, increased amino acid accumulation was found to be correlated with improved plant–water relations, showing the significant role of lysine in improving cellular water relations and osmotic adjustment. Among the studied amino acids, GB and proline are osmotica that have well-known roles in cellular osmotic adjustment [58], maintaining plant–water relations and improving plant photosynthetic efficiency, thereby obtaining better growth and grain yield under reduced irrigation [103,126]. Similarly, glutamate has stress ameliorating effects as the precursor of proline, and increases water stress tolerance by playing roles in growth and yield increments through osmotic adjustment and improvements in plant–water relations [6,7,11]. Accumulation of osmotica, such as certain amino acids, decreases cell osmotic potential, resulting in more water uptake and maintenance of cell turgidity [127,128] necessary for better photosynthetic efficiency through better stomatal regulations associated with turgid guard cells [129], leading to improvement in biomass production and grain yield [130]. In the present findings, Zn-Lys exogenous application significantly increased the accumulation of varying amino acids under limited water supply and in normal irrigation that resulted in more water uptake. This water uptake, due to amino acid accumulation and maintenance of leaf hydraulic conductance, resulted in improved cellular turgidity for better growth with less negative values of cellular osmotic potential in Zn-Lys-applied water-stressed plants than the non-treated ones. In the cases of Pro, GB and Glu, an increase was found in both maize cultivars when grown under deficit irrigation conditions in comparison with well-irrigated plants, correlating better maintenance of LRWC, *g_s_*, and *A*/*E*. So, the stress tolerance induction in maize cultivars for better yield and growth by maintaining better water relations is the function of accumulated osmotica, such as Pro and GB, leading to better *g_s_* and *A*/*E*. So, the improvement in leaf photosynthetic efficiency after foliar-applied Zn-Lys correlates with improved water relations which might be due to better uptake of water from the soil, leading to turgid guard cells, with better stomatal openings and exchange of gasses. In earlier studies it is reported that foliar-applied Zn-chelated Lys resulted in improved plant photosynthetic efficiency in radish and wheat plants [54,101], and therefore better final yield.

Better photosynthesis results in better performance of stomatal factors and depends on the light-capturing efficiency of leaf photosynthetic pigments such as T. Chl., Chl. *a* and *b* leaf carotenoid contents under water deficit conditions [95,131,132]. The light capturing efficiency is severely disturbed under water-stress [133]. In the present study the leaf photosynthetic pigment of maize plant decreased under deficit irrigation, which is positively correlated with a decrease in leaf photosynthetic efficiency. Foliar fertigation with Zn-Lys significantly reduced the negative impacts of water-stress on leaf photosynthetic pigments in both maize cultivars in parallel with gas exchange attributes, showing a positive correlation of light-capturing efficiency and leaf photosynthetic efficiency. So, the increased photosynthetic efficiency of maize plants when grown under deficit irrigation seems to be due to an increment in leaf photosynthetic pigments along with improvement in plant–water relations that correlates with the leaf photosynthetic efficiency in the present findings. Zn has a major role in chlorophyll biosynthesis, demonstrated in plants grown in Zn deficit soil which had a smaller amount of chlorophyll pigments compared to plants grown in Zn-enriched soil [134,135]. Plant photosynthetic rate is linked with the contents of leaf photosynthetic pigments [136] that affect the growth rate of plants and final yield. Chlorophyll *a* and T. Chl. contents are positively linked with the leaf photosynthetic rate [137]. This can be directly linked with the findings of the present study where fertigation of Zn improved the leaf chlorophyll content and photosynthetic efficiency in water-stressed maize plants that is also linked with improved plant biomass accumulation and grain yield, which is likely the function of better Zn availability having a role in chlorophyll biosynthesis [134,135].

Increased accumulation of ROS at cellular levels under adverse environmental conditions is a common phenomenon that results in damages to cellular membranes and other macromolecules. The levels of damages due to over-produced ROS are associated with MDA accumulation [138]. To counteract the negative impacts of overly produced ROS, a well-developed antioxidative defense mechanism has evolved in plants constituting the antioxidative enzyme [139,140] and non-enzymatic metabolic compounds. However, the efficiency of antioxidation is plant-species- and cultivar specific [100]. The same was found in the present study where more accumulation of MDA in genotype Pearl meant it showed less efficiency to scavenge overly produced ROS. In the present study, fertigation of Zn-Lys significantly improved the antioxidative defense mechanism in both maize genotypes with a significant decrease in MDA levels. This improvement in the mechanism of antioxidation might be the function of increased levels of non-enzymatic antioxidative compounds such as AsA, phenolics, flavonoids and carotenoids and the increased activities of SOD, POD, CAT and APX, which result in less leaky membranes and maintenance of better cellular turgidity [141], necessary for better growth [142]. Under water deficit conditions, increased levels of ROS disturb the cellular membranes by lipid peroxidation and disturbs the leaf or root hydraulic conductance by reducing the expression of specialized water channels known as aquaporins (AQPs) [120,121]. In a way, this mechanism is overall harmful as it disturbs the leaf photosynthetic efficiency by decreasing the leaf *g_s_* and *E*, affecting the leaf photosynthetic rate [121]. In maize plants of both cultivars oxidative stress damages are clear in terms of MDA accumulation as well as disturbed plant–water relations and photosynthetic efficiency. In plants supplied with Zn-Lys, reduced oxidative damages are clear due to less MDA accumulation and improved water relations and photosynthetic efficiency, which may be the function of a decline in ROS accumulation with increased antioxidant enzymes activities, resulting in increased expressions of AQPs leading to better maintenance of leaf hydraulic conductance [120] and photosynthetic efficiency for better growth and yield. Moreover, low availability of water disturbs plant photosynthetic rate due to disturbances in the electron transport chain by disturbing the electron transport rate (ETR), causing the generation of ROS in excessive amounts [143] that react with the lipid membranes and cause damages resulting in loss of cellular osmotic potential [144]. Zinc is also involved in the functioning of many antioxidative enzymes by playing a role as a co-factor. It also has a role in the protection of cells from oxidative damages, enhances membrane stabilization, inhibits the activity of NADPH-oxidase (a pro-oxidant enzyme) and induces synthesis of metallothionein [145]. Furthermore, Zn is a structural component of SOD that helps in the formation of hydrogen peroxide from superoxide and molecular oxygen [146,147]. Hydrogen peroxide is an ROS that has both positive and negative influences depending on its cellular levels. At high levels it causes lipid peroxidation but in low concentrations it functions as cellular signaling molecule and plays a role in improving water relations by maintaining the plant hydraulic conductivity. Aroca et al. [148] and Sheripova et al. [149] reported that low concentrations of H_2_O_2_ activated expressions of AQPs in maize and barley and improved plant–water relations. However, a high concentration of H_2_O_2_ inactivated the AQPs in maize [150,151]. In the present study Zn-Lys application significantly improved the activities and contents of non-enzymatic antioxidants, and antioxidant enzymes correlated with improved plant–water relations and reduced H_2_O_2_ content. Improved antioxidative defense mechanisms might found effective in reducing the cellular H_2_O_2_ content and in maintaining better cellular water relations in an AQPs-dependent way, that might result in better biomass accumulation and seed yield of maize plants under deficit irrigation. Along with increments in different enzymatic and non-enzymatic antioxidants, deficit irrigation significantly decreased leaf phenolic levels in both maize cultivars. Phenolic compounds have the ability to counteract overly produced ROS to reduce its damages on cellular membranes [152,153]. Fertigation with different Zn levels as Zn-Lys improved the leaf phenolic contents in maize. The increment in leaf phenolic accumulation might be the role of lysine in the production of shikimate dehydrogenase enzyme that is involved in the biosynthesis of phenolic compounds [154]. Among others, leaf AsA levels were also improved in water-stressed plants of both maize cultivars. The role of AsA as a non-enzymatic antioxidant is well documented [155,156]. Exogenously applied Zn as Zn-Lys increased the leaf AsA levels in maize plants of both genotypes. This shows the role of Zn-Lys in the improvement of antioxidative mechanism efficiency through improving the levels of TPC and AsA. Furthermore, in the present findings an increment in leaf proline accumulation is linked with an improvement in leaf–water relations and antioxidative defense mechanisms. Proline acts as an osmotica and as an antioxidative compound [157]. So, this increased accumulation of proline due to foliar-applied Zn-Lys reveals its role in maintaining cellular osmotic adjustment and in antioxidative defense mechanisms [157,158,159]. In earlier studies, it was found that micronutrients’ fertigation chelated with amino acids improved the antioxidative defense mechanism with decreased lipid production when grown in deficit irrigation compared with their respective chemical fertilizers [35]. Moreover, the improvement in antioxidative defense mechanisms is positively correlated with the improved content of photosynthetic pigment, showing less damage to chloroplastic membranes, and resulted in better light capturing ability, leading to better photosynthetic efficiency [160]. 

The better stress tolerance of maize plants regarding growth and seed yield results in better nutrient uptake which plays and important role in different metabolic activities such as water relations, plant photosynthetic efficiency, assimilation and antioxidative defense mechanisms [161,162,163]. A reduced uptake was found in different nutrients including Mg, K, Ca, P, N and Zn due to water shortage in plants under an irrigation deficit [161]. However, fertigation of Zn-Lys as a foliar spray enhanced the uptake of nutrients, which had significant roles in stabilizing the cellular redox potential and osmotic potential [164] and in the structure and control of metabolic enzyme activities [58,158]. Improvement in the uptake of nutrients positively correlated with improved plant–water relations, chlorophyll content and antioxidative defense mechanisms. The increased uptake in nutrient might be due to accumulation of osmotica, such as different amino acids [35,163], because amino acids act as osmotica and regulate plant–water relations [103,141,165]. 

## 5. Conclusions

Zn-Lys fertigation was found to be helpful in reducing the negative impacts of deficit irrigation on plant biomass and seed yield of both maize genotypes, which was found to be associated with its involvement in improving leaf photosynthetic efficiency, by maintaining better plant–water relations by cellular osmotic adjustment. Moreover, the % increase in seed yield and biomass of maize plants of both cultivars under deficit irrigation due to Zn-Lys foliar spray was larger in comparison with non-stressed ones, showing the drought tolerance induction role of foliar-applied Zn-Lys. Improvements in leaf photosynthetic pigments, reduced lipid peroxidation due to enhanced antioxidations and better uptake of nutrients were also found due to Zn-Lys foliar spray. The improvement in drought tolerance of maize plants due to fertigation with Zn-Lys might also be due to the improved leaf hydraulic conductance in antioxidative defense mechanisms. Exogenously-applied Zn-Lys significantly improved the plant Zn and Lys levels contents, which was helpful for the induction of drought tolerance and may improve the nutritional quality of seeds by improving their contents (this topic requires further study), which could be beneficial to counteract Zn and Lys deficiency in humans. Therefore, recommendations should be made in the use of Zn-Lys as a foliar spray to induce water deficit stress tolerance and to fulfill maize Zn and Lys deficiency for better seed yield and nutritional quality under limited water supply.

## Figures and Tables

**Table 1:** Leaf Glu, Meth, Lys, Asp, proline and GB contents of two maize cultivars foliar-supplied with different levels of Zn-Lys when grown under different irrigation levels (Mean ± SE; *n* = 4).

		**Asp (mg Kg^−1^ FW)**		**Lys (mg Kg^−1^ FW)**		**Meth (mg Kg^−1^ FW)**	
		**Non-Stress**	**Water Stress**	**Non-Stress**	**Water Stress**	**Non-Stress**	**Water Stress**
Pearl	NS	4.80 ± 0.20 ^d^	10.23 ± 0.44 ^d^	10.34 ± 1.61 ^d^	6.45 ± 1.49 ^c^	9.11 ± 0.36 ^d^	13.00 ± 0.45 ^d^
	WS	4.91 ± 0.19 ^d^	10.75 ± 0.53 ^c^	10.37 ± 1.64 ^d^	6.75 ± 1.51 ^c^	9.93 ± 0.39 ^c^	13.21 ± 0.44 ^d^
	0.25% Zn-Lys	6.38 ± 0.19 ^c^	12.32 ± 0.41 ^b^	15.21 ± 1.60 ^c^	9.00 ± 1.56 ^b^	11.21 ± 0.44 ^b^	14.00 ± 0.45 ^c^
	0.5% Zn-Lys	6.67 ± 0.20 ^b^	13.75 ± 0.47 ^a^	16.33 ± 1.72 ^b^	9.33 ± 1.59 ^b^	11.90 ± 0.47 ^a^	14.50 ± 0.47 ^b^
	0.75% Zn-Lys	6.91 ± 0.19 ^a^	13.98 ± 0.39 ^a^	17.00 ± 1.77 ^a^	10.00 ± 1.62 ^a^	12.07 ± 0.48 ^a^	15.00 ± 0.48 ^a^
MMRI	NS	4.70 ± 0.18 ^x^	12.00 ± 0.48 ^y^	9.88 ± 1.59 ^y^	5.11 ± 1.52 ^y^	8.23 ± 0.34 ^y^	12.50 ± 0.40 ^y^
	WS	4.85 ± 0.19 ^x^	11.92 ± 0.47 ^y^	10.19 ± 1.60 ^y^	5.37 ± 1.53 ^y^	8.11 ± 0.32 ^y^	12.65 ± 0.45 ^y^
	0.25% Zn-Lys	5.79 ± 0.19 ^w^	13.23 ± 0.44 ^x^	14.00 ± 1.67 ^x^	7.75 ± 1.50 ^x^	10.34 ± 0.41 ^x^	13.45 ± 0.45 ^x^
	0.5% Zn-Lys	5.97 ± 0.19 ^vw^	14.00 ± 0.47 ^w^	15.76 ± 1.71 ^w^	8.90 ± 1.60 ^w^	10.98 ± 0.43 ^w^	14.04 ± 0.48 ^w^
	0.75% Zn-Lys	6.18 ± 0.19 ^v^	15.11 ± 0.40 ^v^	17.00 ± 1.72 ^v^	9.25 ± 1.63 ^v^	11.33 ± 0.45 ^v^	14.44 ± 0.49 ^v^
LSD 5%		0.24		0.35		0.26	
		**Glu (mg Kg^−1^ FW)**		**Proline (µmol g^−1^ FW)**		**GB (µg g^−1^ FW)**	
		**Non-Stress**	**Water Stress**	**Non-Stress**	**Water Stress**	**Non-Stress**	**Water Stress**
Pearl	NS	18.31 ± 0.73 ^e^	22.34 ± 0.81 ^e^	14.33 ± 0.57 ^b^	28.47 ± 1.33 ^c^	5.21 ± 0.20 ^d^	8.09 ± 0.32 ^e^
	WS	18.62 ± 0.74 ^d^	22.89 ± 0.83 ^d^	14.21 ± 0.56 ^b^	28.87 ± 1.35 ^c^	4.99 ± 0.20 ^e^	8.23 ± 0.32 ^d^
	0.25% Zn-Lys	20.11 ± 0.80 ^b^	23.89 ± 0.91 ^c^	15.11 ± 0.60 ^ab^	36.37 ± 1.45 ^b^	5.81 ± 0.23 ^c^	10.24 ± 0.41 ^c^
	0.5% Zn-Lys	20.87 ± 0.83 ^a^	24.56 ± 0.92 ^b^	15.37 ± 0.61 ^ab^	37.11 ± 1.48 ^ab^	6.11 ± 0.24 ^b^	10.87 ± 0.43 ^b^
	0.75% Zn-Lys	19.88 ± 0.79 ^c^	25.00 ± 0.95 ^a^	15.98 ± 0.63 ^a^	38.23 ± 1.52 ^a^	6.65 ± 0.26 ^a^	11.09 ± 0.44 ^a^
MMRI	NS	18.33 ± 0.73 ^z^	21.47 ± 0.81 ^z^	13.33 ± 0.53 ^x^	30.37 ± 1.29 ^x^	5.17 ± 0.20 ^y^	9.02 ± 0.36 ^z^
	WS	18.78 ± 0.75 ^y^	21.89 ± 0.83 ^y^	13.97 ± 0.55 ^wx^	30.87 ± 1.31 ^x^	5.26 ± 0.21 ^y^	9.33 ± 0.37 ^y^
	0.25% Zn-Lys	20.17 ± 0.80 ^x^	23.17 ± 0.88 ^x^	15.22 ± 0.60 ^vw^	35.25 ± 1.41 ^w^	6.11 ± 0.24 ^x^	11.21 ± 0.44 ^x^
	0.5% Zn-Lys	20.89 ± 0.83 ^v^	23.98 ± 0.91 ^w^	15.97 ± 0.63 ^v^	36.13 ± 1.44 ^vw^	6.39 ± 0.25 ^w^	11.76 ± 0.47 ^w^
	0.75% Zn-Lys	20.67 ± 0.82 ^w^	24.67 ± 0.92 ^v^	16.28 ± 0.65 ^v^	37.00 ± 1.47 ^v^	6.78 ± 0.27 ^v^	12.30 ± 0.47 ^v^
LSD 5%		0.14		1.37		0.12	

NS = no spray; WS = water spray; Zn-Lys = Zn-lysine; GB = glycine betaine; Glu = glutamate; Meth = methionine; Lys = lysine; Asp = aspartate. Means with different letters in superscript in a column differ significantly; a, b, c… and x, y, z… refer to cultivars Peal and MMRI, respectively, under both water regimes.

**Table 2:** Shoot N, P, K, Ca, Mg, Fe and Zn contents of two maize genotypes foliar-supplied with different levels of Zn-Lys when grown under different irrigation levels (Mean ± SE; *n* = 4).

		**N S (mg g^−1^ DW)**		**P S (mg g^−1^ DW)**		**K S (mg g^−1^ DW)**		**Ca S (mg g^−1^ DW)**	
		**Non-Stress**	**Water Stress**	**Non-Stress**	**Water Stress**	**Non-Stress**	**Water Stress**	**Non-Stress**	**Water Stress**
Pearl	NS	37.00 ± 1.48 ^b^	25.77 ± 1.07 ^c^	4.97 ± 0.19 ^c^	2.66 ± 0.10 ^c^	33.21 ± 1.31 ^b^	20.61 ± 0.90 ^b^	4.41 ± 0.17 ^c^	1.91 ± 0.07 ^d^
	WS	37.21 ± 1.48 ^b^	26.00 ± 1.07 ^c^	5.01 ± 0.20 ^c^	2.70 ± 0.10 ^c^	33.00 ± 1.32 ^b^	20.81 ± 0.89 ^b^	4.50 ± 0.18 ^c^	2.00 ± 0.08 ^d^
	0.25% Zn-lys	39.88 ± 1.59 ^a^	29.65 ± 1.18 ^b^	5.32 ± 0.21 ^b^	3.01 ± 0.12 ^b^	33.47 ± 1.33 ^a^	23.25 ± 0.91 ^a^	5.29 ± 0.21 ^b^	2.79 ± 0.11 ^c^
	0.5% Zn-lys	39.97 ± 1.59 ^a^	30.35 ± 1.19 ^a^	5.47 ± 0.21 ^a^	3.16 ± 0.12 ^a^	33.77 ± 1.35 ^a^	24.00 ± 0.92 ^a^	5.57 ± 0.22 ^a^	3.25 ± 0.12 ^a^
	0.75% Zn-lys	39.72 ± 1.58 ^a^	29.49 ± 1.18 ^b^	5.38 ± 0.21 ^ab^	3.07 ± 0.12 ^ab^	33.38 ± 1.33 ^a^	24.00 ± 0.91 ^a^	5.21 ± 0.20 ^b^	2.91 ± 0.10 ^b^
MMRI	NS	38.11 ± 1.52 ^x^	27.88 ± 1.11 ^y^	4.45 ± 0.17 ^y^	2.14 ± 0.08 ^x^	32.11 ± 1.28 ^w^	21.51 ± 0.86 ^w^	4.78 ± 0.19 ^x^	2.18 ± 0.09 ^x^
	WS	38.32 ± 1.53 ^x^	27.50 ± 1.12 ^z^	4.39 ± 0.17 ^y^	2.08 ± 0.08 ^x^	32.31 ± 1.29 ^w^	21.71 ± 0.86 ^w^	4.70 ± 0.18 ^x^	2.20 ± 0.08 ^x^
	0.25% Zn-lys	39.81 ± 1.59 ^w^	31.00 ± 1.18 ^v^	5.38 ± 0.21 ^w^	3.25 ± 0.12 ^vw^	34.21 ± 1.36 ^v^	23.61 ± 0.94 ^v^	5.37 ± 0.21 ^w^	3.11 ± 0.11 ^v^
	0.5% Zn-lys	40.22 ± 1.60 ^v^	30.50 ± 1.12 ^w^	5.49 ± 0.22 ^v^	3.18 ± 0.12 ^w^	34.67 ± 1.38 ^v^	24.07 ± 0.96 ^v^	5.59 ± 0.22 ^v^	2.90 ± 0.12 ^w^
	0.75% Zn-lys	39.79 ± 1.59 ^w^	30.00 ± 1.18 ^x^	5.23 ± 0.20 ^w^	3.35 ± 0.12 ^v^	34.10 ± 1.36 ^v^	23.50 ± 0.94 ^v^	5.30 ± 0.21 ^w^	2.95 ± 0.11 ^w^
LSD at 5% level		0.36		0.11		0.90		0.13	
		**Mg S (mg g^−1^ DW)**		**Fe S (mg g^−1^ DW)**		**Zn S (mg g^−1^ DW)**			
		**Non-Stress**	**Water Stress**	**Non-Stress**	**Water Stress**	**Non-Stress**	**Water Stress**		
Pearl	NS	2.15 ± 0.08 ^c^	1.08 ± 0.04 ^b^	22.31 ± 0.89 ^c^	16.06 ± 0.64 ^c^	24.11 ± 0.96 ^d^	16.00 ± 0.64 ^d^		
	WS	2.13 ± 0.08 ^c^	1.06 ± 0.04 ^b^	22.11 ± 0.88 ^c^	15.86 ± 0.63 ^c^	24.00 ± 0.96 ^d^	15.89 ± 0.63 ^d^		
	0.25% Zn-lys	3.35 ± 0.12 ^a^	1.90 ± 0.08 ^a^	29.11 ± 1.16 ^b^	22.86 ± 0.91 ^b^	36.00 ± 1.33 ^c^	22.15 ± 1.00 ^c^		
	0.5% Zn-lys	3.29 ± 0.13 ^a^	1.95 ± 0.08 ^a^	29.73 ± 1.18 ^a^	23.48 ± 0.93 ^a^	38.00 ± 1.36 ^b^	23.12 ± 1.03 ^b^		
	0.75% Zn-lys	3.04 ± 0.12 ^b^	1.97 ± 0.07 ^a^	29.00 ± 1.16 ^b^	22.75 ± 0.91 ^b^	40.00 ± 1.36 ^a^	23.94 ± 1.04 ^a^		
MMRI	NS	2.47 ± 0.09 ^x^	1.40 ± 0.05 ^x^	24.33 ± 0.97 ^x^	18.08 ± 0.72 ^x^	26.11 ± 1.04 ^z^	18.00 ± 0.72 ^x^		
	WS	2.40 ± 0.09 ^x^	1.33 ± 0.05 ^x^	24.19 ± 0.96 ^x^	17.94 ± 0.71 ^x^	26.07 ± 1.04 ^y^	17.96 ± 0.71 ^x^		
	0.25% Zn-lys	3.21 ± 0.12 ^w^	2.14 ± 0.08 ^w^	27.19 ± 1.08 ^w^	20.94 ± 0.83 ^w^	38.00 ± 1.32 ^x^	25.06 ± 1.00 ^w^		
	0.5% Zn-lys	3.48 ± 0.13 ^v^	2.41 ± 0.09 ^v^	27.81 ± 1.11 ^v^	21.56 ± 0.86 ^v^	40.00 ± 1.33 ^w^	25.27 ± 1.01 ^w^		
	0.75% Zn-lys	3.13 ± 0.12 ^w^	2.06 ± 0.08 ^w^	27.00 ± 1.08 ^w^	20.75 ± 0.83 ^w^	41.00 ± 1.35 ^v^	25.78 ± 1.03 ^v^		
LSD at 5% level		0.11		0.21		0.28			

NS = no spray; WS = water spray; Zn-Lys = Zn-lysine; N S = shoot N; P S = shoot P; Mg S = shoot Mg; Ca S = shoot Ca; K S = shoot K; Fe S = shoot Fe; Zn S = shoot Zn. Means with different superscript letters in a column differ significantly; a, b, c… and x, y, z… refer to cultivars Peal and MMRI, respectively, under both water regimes.

**Table 3:** Spearman’s correlation coefficients of growth, photosynthetic attributes, water relations, antioxidants, amino acids and nutrient contents with yield parameters.

Variables	SFW	SDW	RFW	RDW	SL	NOL	GY/Plant	100 GW	NG/cob
SFW	**1**	0.961 ***	0.942 ***	0.960 ***	0.924 ***	0.924 ***	0.924 ***	0.913 ***	0.874 ***
SDW	0.961 ***	**1**	0.991 ***	0.989 ***	0.989 ***	0.917 −***	0.942 ***	0.955 ***	0.938 ***
RFW	0.942 ***	0.991 ***	**1**	0.989 ***	0.987 ***	0.907 ***	0.953 ***	0.966 ***	0.956 ***
RDW	0.960 ***	0.989 ***	0.989 ***	**1**	0.985 ***	0.917 ***	0.946 ***	0.956 ***	0.942 ***
SL	0.924 ***	0.989 ***	0.987 ***	0.985 ***	**1**	0.893 ***	0.924 ***	0.947 ***	0.943 ***
NOL	0.924 ***	0.917 ***	0.907 ***	0.917 ***	0.893 ***	**1**	0.961 ***	0.951 ***	0.929 ***
GY/plant	0.924 ***	0.942 ***	0.953 ***	0.946 ***	0.924 ***	0.961 ***	**1**	0.994 ***	0.980 ***
100 GW	0.913 ***	0.955 ***	0.966 ***	0.956 ***	0.947 ***	0.951 ***	0.994 ***	**1**	0.991 ***
NG/cob	0.874 ***	0.938 ***	0.956 ***	0.942 ***	0.943 ***	0.929 ***	0.980 ***	0.991 ***	**1**
Chl. *a*	0.919 ***	0.907 ***	0.892 ***	0.906 ***	0.881 ***	0.984 ***	0.947 ***	0.936 ***	0.915 ***
Chl. *b*	0.927 ***	0.891 ***	0.867 ***	0.890 ***	0.871 ***	0.950 ***	0.898 ***	0.881 ***	0.861 ***
T Chl.	0.929 ***	0.910 ***	0.892 ***	0.909 ***	0.886 ***	0.983 ***	0.941 ***	0.928 ***	0.908 ***
Chl. *a/b*	0.199 ns	0.278 *	0.299 *	0.259 *	0.252 ns	0.360 **	0.395 **	0.410 **	0.408 **
*A*	0.880 ***	0.865 ***	0.856 ***	0.874 ***	0.843 ***	0.970 ***	0.936 ***	0.925 ***	0.912 ***
*E*	0.862 ***	0.876 ***	0.889 ***	0.888 ***	0.868 ***	0.935 ***	0.967 ***	0.957 ***	0.961 ***
*C_i_*	0.890 ***	0.922 ***	0.932 ***	0.926 ***	0.912 ***	0.969 ***	0.986 ***	0.985 ***	0.981 ***
*g_s_*	0.897 ***	0.945 ***	0.963 ***	0.948 ***	0.935 ***	0.938 ***	0.988 ***	0.994 ***	0.991 ***
*A*/*E*	0.728 ***	0.726 ***	0.669 ***	0.708 ***	0.718 ***	0.828 ***	0.724 ***	0.736 ***	0.719 ***
*A*/*g_s_*	−0.563 ***	−0.678 ***	−0.738 ***	−0.668 ***	−0.686 ***	−0.541 ***	−0.697 ***	−0.721 ***	−0.741 ***
*C_i_*/*C_a_*	0.890 ***	0.922 ***	0.932 ***	0.926 ***	0.912 ***	0.969 ***	0.986 ***	0.985 ***	0.981 ***
WP	−0.889 ***	−0.927 ***	−0.928 ***	−0.924 ***	−0.922 ***	−0.956***	−0.964 ***	−0.972 ***	−0.961 ***
SP	−0.760 ***	−0.852 ***	−0.877 ***	−0.861 ***	−0.876 ***	−0.864 ***	−0.900 ***	−0.921 ***	−0.931 ***
PP	0.940 ***	0.920 ***	0.895 ***	0.904 ***	0.885 ***	0.963 ***	0.942 ***	0.935 ***	0.903 ***
LRWC	0.766 ***	0.850 ***	0.854 ***	0.827 ***	0.855 ***	0.895 ***	0.913 ***	0.932 ***	0.932 ***
SOD	−0.430 ***	−0.590 ***	−0.658 ***	−0.588 ***	−0.620 ***	−0.512 ***	−0.669 ***	−0.698 ***	−0.745 ***
POD	−0.390 **	−0.561 ***	−0.626 ***	−0.569 ***	−0.617 ***	−0.331 *	−0.500 ***	−0.541 ***	−0.590 ***
CAT	−0.409 **	−0.550 ***	−0.635 ***	−0.576 ***	−0.598 ***	−0.407 **	−0.585 ***	−0.612 ***	−0.679 ***
Apx	0.905 ***	0.889 ***	0.875 ***	0.890 ***	0.864 ***	0.971 ***	0.928 ***	0.921 ***	0.898 ***
AsA	−0.559 ***	−0.727 ***	−0.768 ***	−0.715 ***	−0.766 ***	−0.495 ***	−0.624 ***	−0.670 ***	−0.712 ***
TPC	0.885 ***	0.938 ***	0.940 ***	0.933 ***	0.939 ***	0.960 ***	0.970 ***	0.980 ***	0.977 ***
MDA	−0.788 ***	−0.874 ***	−0.879 ***	−0.854 ***	−0.884 ***	−0.893 ***	−0.913 ***	−0.937 ***	−0.943 ***
H_2_O_2_	−0.821 ***	−0.905 ***	−0.904 ***	−0.885 ***	−0.915 ***	−0.908 ***	−0.911 ***	−0.938 ***	−0.938 ***
Asp	−0.647 ***	−0.773 ***	−0.830 ***	−0.802 ***	−0.814 ***	−0.658 ***	−0.790 ***	−0.817 ***	−0.864 ***
Lys	0.932 ***	0.877 ***	0.864 ***	0.883 ***	0.841 ***	0.965 ***	0.927 ***	0.903 ***	0.871 ***
Meth	−0.355 **	−0.544 ***	−0.622 ***	−0.560 ***	−0.608 ***	−0.417 **	−0.593 ***	−0.639 ***	−0.708 ***
Glut	−0.475 ***	−0.635 ***	−0.704 ***	−0.643 ***	−0.673 ***	−0.480 ***	−0.651 ***	−0.687 ***	−0.738 ***
Pro	−0.683 ***	−0.794 ***	−0.849 ***	−0.814 ***	−0.820 ***	−0.701 ***	−0.832 ***	−0.850 ***	−0.883 ***
GB	−0.602 ***	−0.725 ***	−0.786 ***	−0.756 ***	−0.764 ***	−0.571 ***	−0.721 ***	−0.746 ***	−0.790 ***
N S	0.849 ***	0.911 ***	0.931 ***	0.907 ***	0.910 ***	0.923 ***	0.975 ***	0.986 ***	0.990 ***
P S	0.889 ***	0.944 ***	0.949 ***	0.947 ***	0.947 ***	0.953 ***	0.972 ***	0.982 ***	0.980 ***
K S	0.835 ***	0.918 ***	0.943 ***	0.920 ***	0.929 ***	0.911 ***	0.961 ***	0.977 ***	0.993 ***
Ca S	0.873 ***	0.918 ***	0.934 ***	0.920 ***	0.913 ***	0.946 ***	0.984 ***	0.989 ***	0.987 ***
Mg S	0.836 ***	0.814 ***	0.806 ***	0.808 ***	0.778 ***	0.937 ***	0.925 ***	0.908 ***	0.881 ***
Fe S	0.906 ***	0.871 ***	0.861 ***	0.863 ***	0.830 ***	0.935 ***	0.925 ***	0.912 ***	0.876 ***
Zn S	0.851 ***	0.787 ***	0.781 ***	0.792 ***	0.742 ***	0.939 ***	0.908 ***	0.876 ***	0.843 ***

***, ** and * = significant at 0.001, 0.01 and 0.05 levels, respectively; ns = non-significant. List of abbreviations: SFW= shoot fresh weight; SDW = shoot dry weight; SL = shoot length; RDW = root dry weight; RFW = root fresh weight; NOL = number of leaves; GY/plant = grain yield per plant; 100 GW= 100 grain weight; NG/cob = number of grains per cob; Chl. *a* = Chlorophyll a; Chl. *b* = chlorophyll b; T Chl.= total chlorophyll; Chl. *a/b* = chlorophyll *a* to *b* ratio; *A* = leaf net photosynthetic rate; *E* = leaf transpiration rate; *C_i_* = leaf intrinsic CO_2_ concentration; *g_s_* = leaf stomatal conductance; *A*/*E* = water use efficiency; *A*/*g_s_* = intrinsic water use efficiency; *C_i_*/*C_a_* = leaf intrinsic CO_2_ ratio to atmospheric CO2; WP = water potential; SP = solute potential; PP = pressure potential; LRWC = leaf relative water content; SOD = superoxide-dismutase; POD = peroxidase; CAT = catalase; Apx = ascorbate peroxidase; AsA-ascorbic acid; TPC = total phenolic content; MDA = malondialdehyde; H_2_O_2_ = hydrogen peroxide; Asp = aspartate; Lys = lysine; Meth = methionine; Glut = Glutamate; Pro = praline; GB = glycine betaine; N S = shoot nitrogen; P S = shoot phosphorous; K S = shoot potassium; Ca S = shoot calcium; Mg S = shoot magnesium; Fe S = shoot iron; Zn S = shoot zinc.

## Data Availability

The data presented in the manuscript is the sole data and no other data is linked with this data.
